# Phase I Metabolic Genes and Risk of Lung Cancer: Multiple Polymorphisms and mRNA Expression

**DOI:** 10.1371/journal.pone.0005652

**Published:** 2009-05-21

**Authors:** Melissa Rotunno, Kai Yu, Jay H. Lubin, Dario Consonni, Angela C. Pesatori, Alisa M. Goldstein, Lynn R. Goldin, Sholom Wacholder, Robert Welch, Laurie Burdette, Stephen J. Chanock, Pier Alberto Bertazzi, Margaret A. Tucker, Neil E. Caporaso, Nilanjan Chatterjee, Andrew W. Bergen, Maria Teresa Landi

**Affiliations:** 1 Division of Cancer Epidemiology and Genetics, National Cancer Institute, National Institutes of Health, Bethesda, Maryland, United States of America; 2 Core Genotyping Facility, Advanced Technology Program, Science Applications International Corporation-Frederick, Inc., National Cancer Institute-Frederick, Frederick, Maryland, United States of America; 3 Department of Occupational and Environmental Health, Clinica del Lavoro ‘L. Devoto’ University of Milan, Milan, Italy; 4 Department of Preventive Medicine, Istituto di Ricovero e Cura a Carattere Scientifico (IRCCS) Ospedale Maggiore Policlinico, Mangiagalli, Regina Elena Foundation, Milan, Italy; 5 Center for Health Sciences, SRI International, Menlo Park, California, United States of America; Dr. Margarete Fischer-Bosch Institute of Clinical Pharmacology, Germany

## Abstract

Polymorphisms in genes coding for enzymes that activate tobacco lung carcinogens may generate inter-individual differences in lung cancer risk. Previous studies had limited sample sizes, poor exposure characterization, and a few single nucleotide polymorphisms (SNPs) tested in candidate genes. We analyzed 25 SNPs (some previously untested) in 2101 primary lung cancer cases and 2120 population controls from the Environment And Genetics in Lung cancer Etiology (EAGLE) study from six phase I metabolic genes, including cytochrome P450s, microsomal epoxide hydrolase, and myeloperoxidase. We evaluated the main genotype effects and genotype-smoking interactions in lung cancer risk overall and in the major histology subtypes. We tested the combined effect of multiple SNPs on lung cancer risk and on gene expression. Findings were prioritized based on significance thresholds and consistency across different analyses, and accounted for multiple testing and prior knowledge. Two haplotypes in *EPHX1* were significantly associated with lung cancer risk in the overall population. In addition, *CYP1B1* and *CYP2A6* polymorphisms were inversely associated with adenocarcinoma and squamous cell carcinoma risk, respectively. Moreover, the association between *CYP1A1* rs2606345 genotype and lung cancer was significantly modified by intensity of cigarette smoking, suggesting an underling dose-response mechanism. Finally, increasing number of variants at *CYP1A1/A2* genes revealed significant protection in never smokers and risk in ever smokers. Results were supported by differential gene expression in non-tumor lung tissue samples with down-regulation of *CYP1A1* in never smokers and up-regulation in smokers from *CYP1A1/A2* SNPs. The significant haplotype associations emphasize that the effect of multiple SNPs may be important despite null single SNP-associations, and warrants consideration in genome-wide association studies (GWAS). Our findings emphasize the necessity of post-GWAS fine mapping and SNP functional assessment to further elucidate cancer risk associations.

## Introduction

Lung cancer is the second most common malignancy and has the highest cancer mortality rate worldwide, with an estimated 161,840 individuals expected to succumb to the disease in 2008 in the US [1]. Tobacco smoking is the dominant causal factor for lung cancer; however, fewer than 20% of cigarette smokers develop the disease [2], suggesting that inherited genetic factors may also be important risk determinants. Genetic variation at tobacco carcinogen metabolizing enzymes may lead to inter-individual differences in the level of internal carcinogenic dose and to differential risk for individuals with similar exposures [3]. For this reason, genes that encode enzymes activating harmful chemicals are suitable candidates for lung cancer susceptibility studies and have been intensively studied [4]. Nevertheless, the available published data generally offer inconsistent results [5], due to population heterogeneity, low sample size, poor characterization of the exposure, and a few polymorphisms tested with low power to address the presence of their joint effects.

Here we addressed these issues in the analysis of candidate genes in phase I metabolism and lung cancer susceptibility, taking advantage of a large sample size and detailed epidemiological and clinical information of the Environment And Genetics in Lung cancer Etiology (EAGLE) study [6]. Furthermore, we integrated results on polymorphisms with data on expression from the same genes and the same subjects, for the first time in the context of a population study of phase I metabolic genes and lung cancer.

We explored the role of 25 single nucleotide polymorphisms (SNPs) covering important genes involved in the activation of carcinogens from cigarette smoking: cytochrome P450s (*CYP1B1*, *CYP1A1*, *CYP1A2*, and *CYP2A6*), microsomal epoxide hydrolase (*EPXH1*), and myeloperoxidase (*MPO*). We included also SNPs not previously analyzed, thus providing wide loci coverage in areas previously understudied.

### Candidate genes

Many of the chemical carcinogens in tobacco smoke are members of the polycyclic aromatic hydrocarbon (PAH) family [7]. Cytochrome P450 enzymes activate PAHs [8] to epoxide intermediates, which are converted by epoxide hydrolase to the carcinogens diol-epoxides that interact with DNA or proteins to form adducts. In human lung for example, Benzo[a]pyrene (B[a]P) - a major carcinogenic constituent in tobacco smoke - is first metabolically activated by **cytochrome P450 1A1** (*CYP1A1*) and **cytochrome P450 1B1** (*CYP1B1*) to form B[a]P-7,8-dihydroepoxide, which is further hydrolyzed by microsomal epoxide hydrolase (*EPHX1*) to (F)-benzo[a] pyrene-trans-7,8-dihydrodiol. This compound is further metabolized by *CYP1B1* to form benzo[a]pyrene-7,8-dihydrodiol-9,10-epoxide [9], the most mutagenic and carcinogenic metabolite. *CYP1A1* and *CYP1B1* are over expressed in a wide range of human cancers, including breast, colon, lung, brain and testicular cancer [10], [11]. Tobacco smoking can induce *CYP1A1* and *CYP1B1* proteins up to 10-fold higher levels, particularly in subjects (about 10% of the general population) that are more sensitive to enzyme induction [12]. Polymorphisms in *CYP1A1* (chr15q24.1) are the most frequently studied in relation to lung cancer [13]–[17], but results are limited to only a few SNPs (rs4646903, rs1048943, and rs1799814) that are more frequent in Asian than in Caucasian populations. Functional studies for these SNPs have predicted an increased catalytic activity and higher levels of hydrophobic DNA adducts [18]. In close proximity and strong linkage disequilibrium with *CYP1A1* is the **cytochrome P450 1A2** (*CYP1A2*) gene, characterized by a similar activity [19]. Our study included 8 SNPs from the *CYP1A1/A2* region not previously studied in case-control studies of lung cancer, and some of these SNPs were not included in the platforms used for recent genome-wide association studies (GWAS) [20]–[24]. The *CYP1B1* gene is located on chr2p22.2 and characterized by at least 178 SNPs (ncbi.nlm.nih.gov/dbSNP), including 4 common SNPs that encode amino acid substitutions at codons 48, 119, 432, and 435. These four common amino acid variants alter catalytic activity depending on the substrate, e.g., increase for estradiol hydroxylation [25] and decrease for B[a]P epoxidation and phenylimidazo-pyridine metabolism [26]. Relatively few studies have reported on *CYP1B1* polymorphisms and lung cancer susceptibility with inconsistent results [27]–[30]. We selected 7 SNPs in *CYP1B1* gene, 6 of which not previously studied in association with lung cancer. **Microsomal epoxide hydrolase** (*EPHX1* gene, chr1q42.12) plays a dual role in the metabolism of PAHs and other environmental pollutants, detoxification and bioactivation depending on the substrate. It hydrolyzes reactive compounds such as arene, alkene, and aliphatic epoxides, which are generated by cytochrome P450 and other phase I enzymes to the corresponding dihydrodiols through the *trans* addition of water [31]. On the other hand, less reactive dihydrodiols from PAHs can be substrates for further transformation into dihydrodiol-epoxides such as the carcinogen benzo[a]pyrene-7,8-diol-9,10 epoxide [32], [33]. *EPHX1* appears to be expressed in all tissues but the highest concentrations have been found in the liver, gonads, kidneys, lungs, and bronchial epithelial cells [34]. According to the NCBI's dbSNP database, 119 SNPs have been identified in the *EPHX1* gene region, 20 of which are part of the HapMap database. Functional expression studies are available on a limited number of these polymorphisms and showed effects on hydrolase activity in both directions [35]–[37]. Few studies have investigated the association between coding *EPHX1* polymorphisms and lung cancer susceptibility, with disparate findings mainly limited to the two non-synonymous SNPs rs1051740 and rs2234922, as reported by Kiyohara *et al.* in their review [38] and in more recent studies [39], [40]. We included 8 SNPs from EPHX1 gene, 7 of which not previously studied in association with lung cancer. The human **cytochrome P450 2A6** (*CYP2A6*) is responsible for the metabolism of different exogenous compounds including nitrosamines, aflatoxin B1, and other xenobiotic substrates [41]. In addition, *CYP2A6* catalyzes nicotine C-oxidation to cotinine, and the subsequent hydroxylation of cotinine to 3-OH-cotinine [42]. Several genetic polymorphisms including point mutations and deletions have been reported and studied in association with lung cancer with conflicting results in populations from different ethnicities [43]–[45]. In particular the polymorphism *CYP2A6* rs1801272 selected for this study, which causes an amino acid change from *Leu* to *His*, has been object of dispute: studies found a protective association with lung cancer and amount of cigarette smoke [46] which has not been consistently replicated. **Myeloperoxidase** (*MPO* gene, chr17q22) is a lysosomal enzyme present in high concentrations in human lung due to recruitment of neutrophils [47], and activates B[a]P [48] as well as aromatic amines [49] in tobacco smoke and generates carcinogen-free radicals [50]. A single base substitution, −463G>A, in the promoter region of *MPO* reduces transcription activity and DNA adduct levels in bronchoalveolar lavages of smokers [51]. These mechanisms have supported protective effects of the *MPO* −463A allele against lung cancer [52]. However, this possible inverse association with lung cancer risk has remained controversial [53]. Therefore, further study of the effects of this *MPO* polymorphism on lung cancer is warranted, and we included this SNP in our selection.

A precise characterization of the smoking exposure is essential to successfully identify molecular mechanisms involved in tobacco-related lung carcinogenesis. The EAGLE study provides detailed characterization of tobacco smoking including quantitative information on total exposure and daily intake of cigarette smoking. Using this information, we evaluated genotype-smoking interactions by likelihood ratio test, and compared the contributions of total exposure (pack-years) and intensity (cigarettes per day) of smoking using the linear-exponential model for smoking excess odds ratio (EOR) [54]. This model takes into account the correlation between the two smoking variables by describing the EOR per pack-year in terms of delivery rate of exposure. Our analyses also included stratified groups based on major lung cancer histology subtypes. Furthermore, we tested whether the overall lung cancer risk was determined by the combined action of multiple SNPs within the same gene, despite possible null effects in single SNP associations. We analyzed multiple SNPs jointly and performed gene haplotype analysis. The information on gene expression was limited to a subgroup of 44 subjects with adenocarcinoma, but can help clarify biological mechanisms behind the measured associations of lung cancer with polymorphisms in phase I metabolic genes. We prioritized our findings based on a low p-value threshold (p-value≤0.01) and consistency across different analyses. In order to address concerns related to multiple testing and *a priori* knowledge considerations, we computed the False Positive Report Probability (FPRP) [55].

## Results

### Gene polymorphism and population characteristics

The 25 SNPs selected from phase I metabolic genes are presented in Table 1. The gene coverage is described in Supplemental Figure S1. All analyses were restricted to subjects with at least a 90% genotype call rate (i.e. 34 subjects were excluded). All 25 SNPs passed the test for Hardy-Weinberg equilibrium genotype proportions among the 2041 controls, with a p-value of 0.05 as the threshold.

**Table 1:** List of studied genes, polymorphisms, and corresponding characteristics.

Chromosome	Gene	dbSNP (a)	SNP Region/Base Change (a)	AminoAcid Change (a)	Minor Allele (b)	MAF (b)
1q42.12	*EPHX1*	rs2854455	IVS1−1464T>C		C	0.251
		rs3766934	IVS1−1409G>T		T	0.097
		rs2292566	Ex3−8G>A	Lys119Lys	A	0.138
		rs2260863	IVS3+114C>G		G	0.326
		rs2234922	Ex4+52A>G	His139Arg	G	0.196
		rs34143170	Ex6+19C>T	His247His	T	0.06
		rs2292568	Ex6−80C>T	Pro284Pro	T	0.042
		rs1051741	Ex8+31C>T	Asn357Asn	T	0.102
2p22.2	*CYP1B1*	rs163077	*12259C>T		T	0.217
		rs9341266	Ex3−1249C>T (3′ UTR)		T	0.06
		rs162562	Ex3+939A>C (3′ UTR)		C	0.157
		rs1800440	Ex3+315A>G	Asn453Ser	G	0.201
		rs162557	−2919C>T (upstream)		T	0.17
		rs162556	−3922T>C (upstream)		C	0.446
		rs10175368	−5329G>A (upstream)		A	0.282
15q24.1	*CYP1A1*	rs2198843	11599 bp 3′ of STP G>C (intergenic)		C	0.17
		rs2606345	IVS1+606T>G		G	0.358
		rs2470893	−4010G>A (upstream)		A	0.204
		rs12441817	−10375A>G (intergenic)		G	0.079
		rs2472297	−12441G>A (intergenic)		A	0.115
		rs2472299	−17961C>T (intergenic)		T	0.321
15q24.1	*CYP1A2*	rs11072508	14967 bp 3′ of STP T>C (intergenic)		C	0.388
		rs4886410	*18214C>G (intergenic)		G	0.383
19q13.2	*CYP2A6*	rs1801272	Ex3−15T>A	Leu160His	A	0.041
17q22	*MPO*	rs2333227	−642G>A (upstream) (aka −463 promoter)		A	0.255

(a) According to SNP500 database.

(b) Minor Allele and Minor Allele Frequency (MAF) are based on EAGLE controls.

Table 2 shows the frequency distributions and lung cancer association estimates for the main covariate, among the 4016 subjects included in the study. Age, sex and residential area were unrelated to case status, since frequency matching on these factors was in the design. As expected, all smoking related variables were associated with lung cancer, with increasing risks by increasing smoking exposures. Recent former smokers (up to 5 years) showed a higher risk for lung cancer compared to the current smokers. This is likely an artifact due to the fact that people typically quit smoking because of pre-clinical symptoms of lung cancer rather than a reflection of increasing risks in those who quit smoking [56]. In the analyses of genetic association we added the covariate “years since quit smoking” to the model, to adjust both for this reverse causation and for the attenuation of the risk over time.

**Table 2:** Characteristics of lung cancer cases and controls from the EAGLE population with genotype call rate ≥90%, and their association with lung cancer status.

Characteristic	Sub-category	Cases		Controls		Association with case:control status
		n	%	n	%	OR [95%CI]#
Sex						(a)
	Males	1563	79.1	1560	76.4	1.0
	Females	412	20.9	481	23.6	0.88 [0.76–1.03]
Age						(b)
	35–39	11	0.6	15	0.7	1.0
	40–44	17	0.9	26	1.3	0.88 [0.33–2.39]
	45–49	50	2.5	67	3.3	1.03 [0.44–2.46]
	50–54	124	6.3	121	5.9	1.39 [0.61–3.18]
	55–59	222	11.2	289	14.2	1.03 [0.46–2.31]
	60–64	337	17.1	356	17.4	1.25 [0.56–2.77]
	65–69	445	22.5	472	23.1	1.24 [0.56–2.75]
	70–74	442	22.4	412	20.2	1.42 [0.64–3.15]
	75–79	327	16.6	283	13.9	1.56 [0.70–3.48]
Area						(c)
	Brescia	261	13.2	240	11.8	1.0
	Milan	1302	65.9	1389	68.1	0.85 [0.71–1.04]
	Monza	133	6.7	111	5.4	1.10 [0.81–1.50]
	Pavia	126	6.4	122	6.0	0.96 [0.71–1.30]
	Varese	153	7.7	179	8.8	0.78 [0.59–1.04]
Smoking status						(d)
	Never	140	7.1	658	32.2	1.0
	Former, >2years	655	33.2	848	41.5	3.98 [3.18–4.98]
	Former, 0.5 to 2 years	188	9.5	30	1.6	34.37 [22.22–53.16]
	Current	980	49.6	501	24.5	11.37 [9.06–14.28]
	Missing	12	0.6	4	0.2	
Cigarettes per day						(d)
	Never	140	7.1	658	32.2	1.0
	<12	233	11.8	519	25.4	2.54 [1.98–3.26]
	12–20	358	18.1	343	16.8	7.00 [5.41–9.05]
	20–25	571	28.9	290	14.2	14.77 [11.37–19.18]
	>25	568	28.8	226	11.1	19.63 [14.98–25.72]
	Missing	105	5.3	5	0.2	
Total pack-years						(d)
	Never	140	7.1	658	32.2	1.0
	<19.5	187	9.5	578	28.3	1.88 [1.45–2.43]
	19.5–36	381	19.3	365	17.9	7.25 [5.61–9.37]
	36–52.5	539	27.3	275	13.5	15.02 [11.54–19.56]
	>52.5	623	31.5	160	7.8	30.79 [23.19–40.86]
	Missing	105	5.3	5	0.2	
Years since quit						(d)
	Current	980	49.6	501	24.5	1.0
	<5	300	15.2	96	4.7	1.49 [1.15–1.93]
	5–15	249	12.6	179	8.8	0.63 [0.50–0.78]
	15–24	180	9.1	260	12.7	0.31 [0.25–0.39]
	>24	114	5.8	343	16.8	0.14 [0.11–0.18]
	Never	140	7.1	658	32.2	0.09 [0.07–0.11]
	Missing	12	0.6	4	0.2	
Histology						
	Adenocarcinomas	809	41.0			
	Squamous cell carcinoma	505	25.6			
	Small cell carcinoma	201	10.2			
	Others	425	21.5			
	Missing	35	1.8			
Total		1975	100.0	2041	100.0	

^#^Two-sided Wald test.

(a) ORs adjusted for age and area.

(b) ORs adjusted for sex and area.

(c) ORs adjusted for sex and age.

(d) ORs adjusted for sex, age and area.

### SNP and lung cancer risk overall and by histology

Table 3 reports results with p_trend_≤0.05 for the main effect associations between each SNP and lung cancer risk overall and by histology. The complete list of results is reported in Supplemental Table S1.

**Table 3:** Polymorphisms associated with risk of lung cancer overall and by histology with a significant trend (in bold) or nominally significant trend (in *italics*).

SNP	Genotype	Controls	Cases	OR (a)	95%CI−	95%CI+	P-value Trend#
All Histologies							
CYP2A6/rs1801272	T/T	1855	1756	1			
	T/A	160	101	0.74	0.55	1.00	
	A/A	4	2	0.26	0.04	1.94	
	T/A+A/A	164	103	0.73	0.54	0.98	
	Trend			0.72	0.54	0.96	*0.026*
Adenocarcinoma							
EPHX1/rs2292568	C/C	1852	680	1			
	C/T	156	86	1.48	1.09	2.01	
	T/T	7	1	0.41	0.04	4.43	
	C/T+T/T	163	87	1.44	1.06	1.96	
	Trend			1.38	1.03	1.85	*0.032*
CYP1B1/rs9341266	C/C	1798	701	1			
	C/T	222	72	0.8	0.59	1.09	
	T/T	12	1	0.14	0.01	1.24	
	C/T+T/T	234	73	0.76	0.56	1.04	
	Trend			0.74	0.55	0.99	*0.046*
CYP1B1/rs162556	T/T	621	205	1			
	T/C	1002	391	1.15	0.92	1.42	
	C/C	400	172	1.34	1.03	1.74	
	T/C+C/C	1402	563	1.2	0.98	1.47	
	Trend			1.16	1.01	1.32	*0.031*
CYP1B1/rs10175368	G/G	1056	430	1			
	G/A	790	297	0.87	0.71	1.05	
	A/A	176	45	0.55	0.38	0.81	
	G/A+A/A	966	342	0.81	0.67	0.97	
	Trend			0.8	0.69	0.93	**0.003**
Squamous Cell Carcinoma							
CYP2A6/rs1801272	T/T	1855	463	1			
	T/A	160	18	0.48	0.27	0.86	
	A/A	4	0	-	-	-	
	T/A+A/A	164	18	0.47	0.27	0.83	
	Trend			0.47	0.27	0.81	**0.007**

(a) ORs were adjusted for age, sex, area, cigarette per day, total pack-years, years since quit smoking.

^#^Two-sided Wald test.

In adenocarcinoma cases only (test for heterogeneity by histology: p_heterog_ = 0.066), the minor allele of *CYP1B1* rs10175368 was significantly protective for lung cancer (OR = 0.8, 95%CI = 0.69–0.93, p_trend_ = 0.003) and a similar protective effect was nominally significant (i.e. p-value≤0.05) for the *CYP1B1* rs9341266 polymorphism. The cumulative number of variants in *CYP1B1* rs9341266 and *CYP1B1* rs10175368 also conferred a significant protection for lung cancer in adenocarcinoma cases only (OR = 0.83, 95%CI = 0.74–0.94, p_trend_ = 0.002; test for heterogeneity by histology: p_heterog_ = 0.058), in concordance with the two results from the single SNP analyses.

The *CYP2A6* rs1801272 polymorphism was significantly associated with a decreased lung cancer risk in squamous cell carcinoma cases (OR = 0.47, 95%CI = 0.27–0.81, p_trend_ = 0.007; test for heterogeneity by histology: p_heterog_ = 0.045). The protective effect was nominally significant in the overall population. Interestingly, the same SNP was significantly associated with a decrease of cigarette smoking intensity in controls (OR = 0.86, 95%CI = 0.78–0.94, p_trend_ = 0.0007).

### Genotype-smoking interaction

We repeated the analyses within subgroups defined by smoking status (never and ever smokers) in all cases and controls and, separately, in adenocarcinoma cases only and all controls (see Table 4 for the single SNP analysis, and Supplemental Table S2 for the joint SNP analysis). The other histology groups included too few never smokers to perform a meaningful analysis.

**Table 4:** Associations between SNPs and lung cancer risk by never/ever smoking status, for significant (in bold) or nominally significant (in *italics*) smoking-genotype interactions.

SNP	Genotype	Never Smokers						Ever Smokers						
		Controls	Cases	OR (a)	95% CI−	95% CI+	P-val Trend	Controls	Cases	OR (b)	95% CI−	95% CI+	P-val Trend#	LH Ratio P-value
All Histologies														
CYP1A1/rs2606345	T/T	262	73	1				579	724	1				
	T/G	300	54	0.68	0.46	1.01		622	769	0.99	0.84	1.18		
	G/G	95	13	0.48	0.25	0.92		170	233	1.18	0.92	1.53		
	T/G+G/G	395	67	0.63	0.43	0.91		792	1002	1.03	0.88	1.21		
	Trend			0.69	0.52	0.91	**0.009**			1.06	0.94	1.19	0.334	**0.005**
CYP1A2/rs11072508	T/T	247	61	1				515	627	1				
	T/G	299	61	0.86	0.58	1.29		664	803	1.04	0.87	1.24		
	G/G	110	17	0.65	0.36	1.18		196	292	1.31	1.02	1.67		
	T/G+G/G	409	78	0.81	0.55	1.18		860	1095	1.1	0.93	1.3		
	Trend			0.82	0.63	1.08	0.157			1.12	1	1.26	*0.055*	*0.038*
CYP1A2/rs4886410	C/C	249	61	1				521	640	1				
	C/G	300	62	0.85	0.57	1.27		665	805	1.04	0.87	1.24		
	G/G	108	17	0.67	0.37	1.22		187	281	1.3	1.01	1.67		
	C/G+G/G	408	79	0.8	0.55	1.17		852	1086	1.09	0.93	1.29		
	Trend			0.83	0.63	1.09	0.175			1.12	0.99	1.25	0.069	*0.047*
CYP2A6/rs1801272	T/T	601	124	1				1254	1632	1				
	T/A	47	16	1.51	0.81	2.79	91	113	85	0.62	0.44	0.87		
	A/A	0	0	-	-	-		4	2	0.26	0.03	1.92		
	T/A+A/A	47	16	1.51	0.81	2.79		117	87	0.61	0.44	0.84		
	Trend			1.51	0.81	2.79	0.191			0.61	0.44	0.84	**0.002**	*0.026*
Adenocarcinoma														
CYP1A1/rs2606345	T/T	262	51	1				579	281	1				
	T/G	300	38	0.68	0.43	1.08		622	286	0.94	0.76	1.17		
	G/G	95	11	0.59	0.29	1.2		170	107	1.39	1.02	1.89		
	T/G+G/G	395	49	0.66	0.43	1.01		792	393	1.03	0.84	1.27		
	Trend			0.74	0.54	1.02	0.066			1.11	0.96	1.29	0.154	*0.022*

(a) ORs were adjusted for age, sex, area.

(b) ORs were adjusted for age, sex, area, cigarette per day, total pack-years, years since quit smoking.

^#^Two-sided Wald test.

Three SNPs in the chr15q24.1 region (*CYP1A1*/*A2*) showed a protective effect for lung cancer among never smokers but a tendency towards increased risk of lung cancer in ever smokers, with a significant genotype-smoking interaction for *CYP1A1* rs2606345 (p_interact_ = 0.005) and a nominally significant genotype-smoking interaction for the two SNPs in *CYP1A2*.

**Figure 1:**
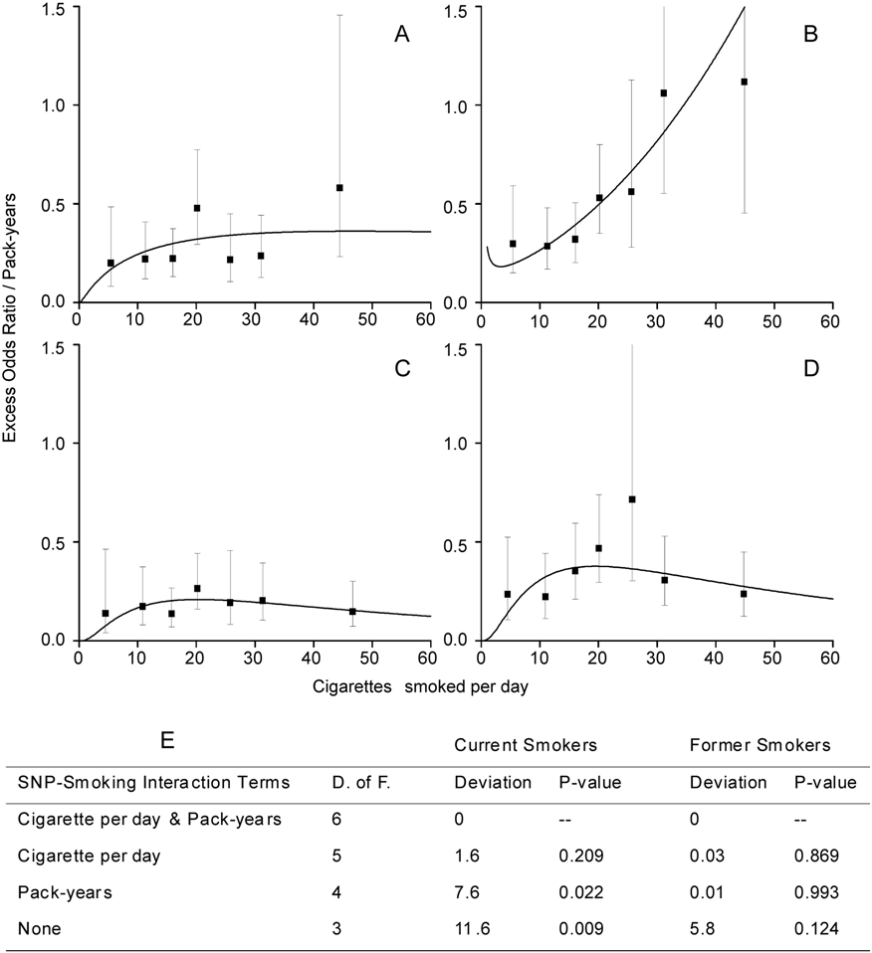
Estimates of the smoking excess odds ratio by *CYP1A1/rs2606345* status. Estimates of the linear slope parameter (EOR per pack-year) and its 95 percent confidence interval within categories of smoking intensity (square symbol) and fitted linear-exponential odds ratio for continuous pack-years and cigarettes per day (solid line) for *CYP1A1* rs2606345. The Figure shows results for T/T genotype in panels A and C, and for *T/G+G/G* genotypes in panels B and D, among current smokers (700 *T/T*+997 *T/G+G/G*) (panels A and B) and former smokers (640 *T/T*+855 *T/G+G/G*) (panels C and D). The table in panel E reports the estimated deviances and p-values for the genotype-smoking interaction among current and former smokers for the model including both interaction terms between the genotype and pack-years and between the genotype and cigarette per day, and for intermediate models including either the interaction term between genotype and pack-years, or the interaction term between genotype and cigarette per day. The significant increase in deviance in current smokers is mainly due to the interaction term of the genotype with cigarettes per day and not with pack-years; the removal of pack-years from the model did not degrade fit relative to the full model (p = 0.209), whereas the removal of cigarettes per day did degrade fit (p = 0.022).

We further explored the significant genotype-smoking interaction in *CYP1A1* rs2606345 by means of the linear-exponential model for smoking excess odds ratio [54], and evaluated whether the variation in smoking risk by genotype resulted from the interaction with smoking intensity or with total pack-years and whether this interaction was present among other categories of smokers such as current or former smokers. Results are shown in Figure 1. The EOR per pack-years in current smokers compared to never smokers (Figure 1A and Figure 1B) increased for increasing number of cigarettes per day, reaching a plateau for subjects carrying the *CYP1A1* rs2606345 homozygote major allele (Figure 1A), and in contrast, increasing exponentially for subjects carrying the *CYP1A1* rs2606345 heterozygote or homozygote minor allele (Figure 1B). The same analysis of EOR/pack-years in former smokers *versus* never smokers (Figure 1C and Figure 1D) similarly showed that the EOR increase for cigarettes per day was lower in homozygote major allele carriers (Figure 1C) than for heterozygote or homozygote minor alleles carriers (Figure 1D), but here EOR/pack-years reached a plateau among both groups of subjects. Panel E in Figure 1 reports the estimated deviances and p-values for the genotype-smoking interaction among current and former smokers for the model including both interaction terms between the genotype and pack-years and between the genotype and cigarettes per day, and for intermediate models including either the interaction term between genotype and pack-years, or the interaction term between genotype and cigarettes per day. The overall genotype-smoking interaction was stronger among current smokers (p_interact_ = 0.009) than among former smokers (p_interact_ = 0.124). Among current smokers, the removal of pack-years from the model did not degrade fit relative to the full model (p = 0.209), whereas the removal of cigarettes per day did degrade fit (p = 0.022), suggesting that the genotype interaction effects resulted from cigarettes per day and not pack-years.

In the joint analysis of multiple SNPs stratified by smoking (Supplemental Table S2), the cumulative number of variants of all 8 SNPs from *CYP1A1* and *CYP1A2* in the chr15q24.1 region conferred a significant overall risk for lung cancer in ever smokers (OR = 1.03, 95%CI = 1.00–1.07, p_trend_ = 0.040) and a borderline protective effect in never smokers (OR = 0.91, 95%CI = 0.84–0.99, p_trend_ = 0.055). The smoking-genotype interaction was highly significant (p_interact_ = 0.006).

In addition, the minor allele of *CYP2A6* rs1801272 showed a significant protective effect in ever smokers, increased risk in never smokers, and a nominally significant genotype-smoking interaction.

### Linkage disequilibrium and haplotype analysis

For genes represented by two or more SNPs, we computed linkage disequilibrium (LD) among controls and haplotype association with lung cancer. The complete results are reported in the Supplemental Text S1 and Figure S2.

Interestingly, the haplotype analysis for the 8 SNPs in *EPHX1* (which were in low LD: r^2^≤0.1 for most SNPs pairs, r^2^ = 0.43 for *EPHX1* rs2234922 and *EPHX1* rs1051741) revealed two haplotypes significantly associated with lung cancer in the overall population: carriers of *TGGCACTC* haplotype had higher risk than non-carriers (freq = 0.01, p-value = 0.010) and carriers of *CGGCGCCT* haplotype had a lower risk than non-carriers (freq = 0.01, p-value = 0.015). In addition, we found similar results in the analysis restricted to adenocarcinoma cases only: *TGGCACTC* (p-value = 0.008) and *CGGCGCCT* (p-value = 0.023). Since the 8 SNPs were in low LD, we also performed a three marker moving window haplotype analysis and found no significant associations between lung cancer and haplotype combinations of three SNPs (see Supplemental Table S3). However, we identified a borderline significant protective association (freq = 0.03, p-value = 0.059) with a three-locus haplotype with a C, G, and T in locus 1, 2 and 8, respectively, which was also contained in the 8 SNP haplotype.

For the 8 SNPs in the chr15q24.1 region, we found two regions of LD, one of modest strength surrounding *CYP1A1*, and a second region 3′ of *CYP1A2* (see Supplemental Figure S2), concordant with the results from HapMap. Haplotype analyses were computed separately for these two LD regions; the *GTAAA* haplotype (freq = 0.07) and the *CGGGG* haplotype (freq = 0.03) were nominally significantly associated with lung cancer risk in never and ever smokers respectively.

### Association between genotype and gene expression

The complete results for the correlation between genotype and gene expression data are reported in Supplemental Table S4. We found that the 8 polymorphisms in the 15q24 chromosomal region had a significant down-regulating effect on mRNA expression for *CYP1A1* gene among the 14 never smokers (δ = −1.51, p-value = 0.007) and showed a trend for up-regulation among the 15 current smokers (δ = 4.95, p-value = 0.078). The 7 polymorphisms in *CYP1B1* were significantly associated with an increase of mRNA expression in *CYP1B1* among the 15 current smokers (δ = 8.99, p-value = 0.004), and not among the 44 subjects overall. For the 8 SNPs in *EPHX1* gene, we observed an overall trend for decreasing expression (δ = −1.20, p-value = 0.096), which was nominally significant among the 15 former smokers (δ = −2.56, p-value = 0.049).

### Multiple testing

FPRP calculations (Table 5) were performed for nominally significant or significant single SNP analysis results. The table shows that all prior probabilities of ≥0.10 had low FPRP values (<0.5).

**Table 5:** False positive report probability.

Gene/SNP	MAF	Controls	Cases	Test	OR(**)	P-value	Power(*)	Prior Probabilities				
								0.5	0.25	0.1	0.01	0.001
CYP2A6/rs1801272	0.041	2019	1859	(a)	0.720	0.026	0.772	*0.033*	*0.092*	**0.233**	0.769	0.971
EPHX1/rs2292568	0.042	2015	767	(b)	1.380	0.032	0.632	*0.048*	*0.132*	**0.313**	0.834	0.981
CYP1B1/rs9341266	0.060	2032	774	(b)	0.741	0.046	0.609	*0.070*	*0.185*	**0.405**	0.882	0.987
CYP1B1/rs162556	0.446	2023	768	(b)	1.156	0.031	0.673	*0.044*	*0.121*	**0.293**	0.820	0.979
CYP1B1/rs10175368	0.282	2022	772	(b)	0.799	0.003	0.907	*0.003*	*0.010*	*0.029*	**0.247**	0.768
CYP2A6/rs1801272	0.041	2019	481	(c)	0.467	0.007	0.926	*0.008*	*0.022*	*0.064*	**0.428**	0.883
CYP1A1/rs2606345	0.358	2028	1866	(d)	0.687	0.005	0.795	*0.006*	*0.019*	*0.054*	**0.384**	0.863
CYP1A2/rs11072508	0.388	2031	1861	(d)	0.822	0.038	0.545	*0.065*	*0.173*	**0.386**	0.873	0.986
CYP1A2/rs4886410	0.383	2030	1866	(d)	0.829	0.047	0.511	*0.084*	**0.216**	**0.453**	0.901	0.989
CYP2A6/rs1801272	0.041	2019	1859	(d)	1.508	0.026	0.757	*0.033*	*0.093*	**0.236**	0.773	0.972
CYP1A1/rs2606345	0.358	2028	774	(e)	0.739	0.022	0.481	*0.044*	*0.121*	**0.291**	0.819	0.979

FPRP values for the nominally significant (p-value<0.05) results from test of main single SNP effects (Table 3) and of SNP-smoking interaction effects (Table 4). FPRP is computed according to the formula α(1−π)/[α(1−π)+(1−β)π], where α and (1−β) are the P-value and Power values reported in the table, and π represents the Prior Probability ranging from 0.001 to 0.5. FPRP values less than 0.2 are in italic, FPRP values between 0.2 and 0.5 are bold, and FPRP values larger than 0.5 are the rest.

(a) Test for main genetic effect among all subjects.

(b) Test for main genetic effect among controls and adenocarcinoma cases.

(c) Test for main genetic effect among controls and squamous carcinoma cases.

(d) Test for gene-smoking interaction among all subjects.

(e) Test for gene-smoking interaction among controls and adenocarcinoma cases.

(*) OR indicates the measured odds ratio for the main genetic effect for tests (a), (b), and (c), and the measured odds-ratio ratio for the gene-smoking interaction effect for tests (d) and (e).

(**) The statistical power to detect the measured OR given a type I error rate of 0.05 was computed by means of the QUANTO software (http://hydra.usc.edu/gxe).

## Discussion

In this large population-based case-control study of lung cancer we have observed that *EPHX1*, *CYP1A1*, *CYP1B1* and *CYP2A6* genes may play a role in lung cancer susceptibility.

Two haplotypes in *EPHX1* compared to all other haplotypes were significantly associated with lung cancer in the overall population and in adenocarcinoma cases only: *TGGCACTC* as a risk factor and *CGGCGCCT* as a protective factor. In addition, we identified a borderline significant protective association with a three-locus haplotype which was also contained in the 8SNP haplotype and was present in approximately 3% of the population. These findings suggest that more than a hundred people in our study carried a three-variant haplotype resulting in a decreased lung cancer risk. The protective effect was even stronger for the smaller number of subjects (1%) who carried a combination of these three SNPs and the remaining 5 SNPs in the 8-locus haplotype. Since the significant associations with lung cancer were based on relatively rare haplotypes, replication will be needed in order to validate this finding. None of the 8 SNPs was significantly associated with lung cancer in the overall population when analyzed separately. This result, if confirmed, demonstrates that the effect of multiple SNPs on lung cancer may be important even if most individual SNPs do not show significant association. This may explain why previously published results, which are based on a limited number of *EPHX1* polymorphisms, were inconsistent. In particular, *EPHX1* rs2234922 has been previously associated both with risk [39] and with protection [57] for lung cancer. This SNP was not associated with lung cancer in our data. Nevertheless it was one of the three SNPs that differentiate the two significant haplotypes reported here. The other two SNPs were *EPHX1* rs1051741, in medium LD with *EPHX1* rs2234922, and *EPHX1* rs2292568, nominally significantly associated with risk of lung adenocarcinoma in our data (see Table 3). We did not find a significant association between *EPHX1* polymorphisms and gene expression. Measurements of epoxide hydrolase activity in lung cancer patients carrying these haplotypes will be needed in order to understand the biological mechanism that underlies this finding.

A group of SNPs from two LD regions in the chr15q24.1 region (*CYP1A1* and *CYP1A2*) showed a protective effect on lung cancer risk among never smokers and a suggestive risk of lung cancer in ever smokers with a significant genotype-smoking interaction for *CYP1A1* rs2606345 and a nominally significant interaction for the two SNPs in *CYP1A2*. This result was confirmed by the multiple SNP analysis stratified by smoking. The cumulative number of variants from *CYP1A1* and *CYP1A2* was in fact associated with a significant risk for lung cancer in ever smokers and a protective effect in never smokers, with a highly significant smoking-genotype interaction. Interestingly, Wang *et al.* [58] recently reported an analogous inverse association between *CYP1A1* rs2606345 and levels of DNA adducts: the variant allele was associated with high level of DNA adducts among women with high PAH exposure and with low level of DNA adducts among women with low PAH exposure. Further, using the linear-exponential model for smoking EOR we found that the difference in smoking effects between the wild type and the variant resulted from the effects of cigarettes per day and not pack-years. This finding suggests that a dose-response mechanism and a saturation effect might underlie the smoking-mediated association between *CYP1A1* and lung cancer risk. The gene expression analysis supported this finding. In fact, the lower expression of *CYP1A1* among never smokers and higher expression among current smokers in association with the SNPs at chr15q24.1 was consistent with the observed protective effect for lung cancer among never smokers and risk among smokers in association with variants in *CYP1A1/A2*.

Our data also showed that the minor allele of *CYP1B1* rs10175368 was significantly protective for adenocarcinoma of the lung (OR = 0.80, 95%CI = 0.69–0.93) and a similar protective effect was observed for the minor allele of *CYP1B1* rs9341266 (r^2^ = 0.30), as well as for the cumulative sum of the two minor alleles. In addition, according to the HapMap database, *CYP1B1* rs10175368 is in LD with 4 other SNPs in the same chromosomal region (rs2551188, rs4646430, rs4646429, and rs10175338, see Supplemental Figure S1B). These 4 SNPs are likely to be characterized by the same protective association. Previous results on *CYP1B1* polymorphisms and lung cancer have been limited to the four non-synonymous SNPs rs10012, rs1056827, rs1056836 and rs1800440 [27]–[30], [59], [60]. None of the reported positive findings have been consistently replicated, except for rs10012, associated with lung cancer risk in two independent studies [28], [30]. Our data on rs1800440 did not show any significant association with lung cancer. The three other non-synonymous SNPs were not evaluated in the current study. However, our SNPs were selected with an attempt to cover other regions of the gene. According to our data, variants other than those in the coding region could alter lung cancer risk. Polymorphisms in *CYP1B1* have been associated with decreased PAH metabolism [26]. The significant protective effect of the *CYP1B1* rs10175368 variant allele could be due to a lower level of smoking carcinogens in subjects carrying the variant allele. We did not find a significant effect on *CYP1B1* gene expression for the two SNPs in *CYP1B1* associated with a protection for adenocarcinoma. However, when we considered all seven polymorphisms in *CYP1B1* together and studied their effect on gene expression, we found a significant increase in *CYP1B1* gene expression among current smokers. The *CYP1B1* gene is known to be highly expressed in lung tissues of lung cancer patients. Our result supports previous findings of *CYP1B1* gene over-expression among current smokers [61] and suggests a possible involvement of *CYP1B1* polymorphisms as a mechanism for differential expression.

The *CYP2A6* rs1801272 polymorphism, which results in an amino acid change from *Leu* to *His*, was significantly associated with a decreased risk for squamous cell carcinoma, a strictly smoking-related malignancy. Interestingly, the same SNP was associated with a decrease in cigarettes per day in controls, confirming a previously hypothesized role of this gene in tobacco smoking addiction [46]. Our report provides the first confirmation of this finding in a population-based sample. In addition, the *A* allele of *CYP2A6* rs1801272 showed a significant protective effect in ever smokers but no effect in never smokers, with a nominally significant genotype-smoking interaction due to the effect of cigarettes per day and not pack-years. The *CYP2A6* gene is characterized by multiple polymorphisms and genomic repetitive elements in the regulatory regions, which make a complete coverage of the gene extremely challenging. Moreover, most variants are very rare in the general population and would not be identifiable even in a large sample size as ours. We genotyped *CYP2A6* rs1801272 (also known as CYP2A6*2) because this SNP is relatively common (4% in our population), has been well characterized in previous functional studies [46], and showed controversial associations with cancer and smoking dependence [43]–[46]. Our findings of an association with both lung cancer risk and tobacco addiction warrant further investigation based on a more complete coverage of this gene.

The size of our population provides unusual power for confirming previously reported associations. Our data do not support proposed associations between lung cancer and *EPHX1* rs2234922, *CYP1B1* rs1800440, and *MPO* rs2333227. The confidence in our significant results was supported by the low FPRP values (see Table 5) observed for prior probabilities of 0.10 or more given the strong prior probabilities of the selected phase I genes being involved in lung cancer risk.

At the time that this project was initiated, there was less genotype data available with which to select SNPs to cover haplotype blocks. Nevertheless, based on the existing SNP500Cancer and comparative assessment of HapMap data, we selected SNPs that represented tagSNPs in the Caucasian population. Although the coverage is inevitably incomplete, we substantially improved the coverage of the selected genes in comparison with previous studies (see Supplemental Figure S1).

Strengths of our study include a population-based design, large sample size with adequate power to detect main gene effect and gene-smoking interaction effect, integrative analysis with gene expression data, and a systematic approach in evaluating the joint effects of multiple SNPs.

Our results are particularly timely in relation to recent GWAS. For example, the significant association between haplotypes in *EPHX1* and lung cancer risk emphasizes that the effect of multiple SNPs may be important despite null associations in single SNP analyses, and should be taken into consideration in GWAS. Similarly, although further study is necessary to confirm the qualitative interaction between smoking and genotype in relation to lung cancer susceptibility for the *CYP1A1* rs2606345, this finding is particularly interesting, considering that this SNP is not included in the HapMap database or in the common platforms used for GWAS, although it is in relatively strong LD with other SNPs in these platforms. This highlights the necessity of fine mapping after GWAS to further elucidate associations with lung cancer risk and tobacco smoking addiction. In conclusion, this study emphasizes the importance of ample coverage of genes in the analysis of genetic susceptibility of cancer, integration with corresponding gene function in the target tissue, and rigorous study design and analytical approach.

## Materials and Methods

### Study population and data collection

A detailed description of the EAGLE study has been recently published [6]. Briefly, the study includes 2101 incident lung cancer cases and 2120 population controls enrolled in the period April 2002–June 2005 in 216 municipalities from the Lombardy region (Italy). Cases were subjects with primary cancer of trachea, bronchus and lung, first diagnosed between April 22, 2002 and February 28, 2005, and admitted to 13 hospitals of the study area. Controls were randomly sampled from population databases, frequency matched to cases by area of residence (5 classes), gender, and age (5-year categories), and contacted through the family physician. All enrolled subjects were Caucasian. Subjects were 35–79 years of age at diagnosis (cases) or at sampling/enrollment for interview (controls). The study participation rates were 86.6% among cases and 72.4% among controls. After signing an Institutional Review Board-approved informed consent form, subjects underwent a computer-assisted personal interview (CAPI) and filled-in a self-administered questionnaire. Biospecimens (blood or buccal rinse from all participants and pathological samples from cases) were collected. Epidemiological information on the 4016 EAGLE subjects with available genotype data and analyzed in this study is described in Table 2.

### SNP selection and genotyping

At the start of the study, SNP assays were selected from those available at the Core Genotyping Facility (CGF) of the Division of Cancer Epidemiology and Genetics (National Cancer Institute), using our own assessment of linkage disequilibrium between the SNPs from HapMap and previous evidence from the literature. The 25 SNPs selected from phase I metabolism genes are presented in Table 1. The gene coverage for *EPHX1*, *CYP1B1*, and *CYP1A1/A2* based on the present version of the HapMap database is described in Supplemental Figure S1. For *CYP2A6* and *MPO* genes, we selected only two SNPs whose association with lung cancer has been debated in previous studies [43]–[45], [52], [53]. Genotyping of the 25 SNPs was done at the CGF with the TaqMan® assay, described at the National Cancer Institute SNP500Cancer website (http://snp500cancer.nci.nih.gov). Genotyping was performed on 4050 EAGLE subjects (those with sufficient DNA samples). Duplicate quality-control samples (2% of the total) showed 100% agreement for all 25 assays.

### Gene expression data

In addition to genotype information, we analyzed mRNA gene expression data from an Affymetrix HG-U133A microarray using fresh tissue samples from a subgroup of adenocarcinoma cases. The original microarray study has been described elsewhere [61]. Here, we analyzed the gene expression data from non-tumor samples of 44 subjects in relation to genotype data from the same subjects, as described in the Statistical analysis section.

### Statistical analysis

Most analyses were implemented and performed using the R-project (version 2.8) statistical package (http://www.r-project.org/index.html). The EOR smoking model was implemented in Epicure (http://www.hirosoft.com/).

#### A. Main effect of genotype

The main effect of the variant genotypes on the risk of lung cancer was estimated by odds ratios and their 95% confidence intervals using unconditional logistic regression analysis. Homozygosity for the more frequent allele among controls was defined as the reference group. We tested for significance using two-sided Wald tests. The trend test for the effect of SNP was conducted by including the SNP variable as continuous in logit scale in the model, and the categorical analysis was performed by treating the SNP variable as three levels categorical variable. Age, sex, geographical location, cumulative smoking dose (pack-years), smoking intensity (cigarettes per day), and quitting smoking (years since quit) were selected as covariates. We performed stratified analyses by smoking status (never/ever) of cases and controls and polytomous logistic regression by the major histology types (adenocarcinoma, squamous cell carcinoma, and small cell carcinoma) of cases. In the analysis by histology, we defined the standard Wald chi-square test statistic using the coefficient estimates derived from a polytomous logistic regression (where the response variable was coded on four levels: controls, adenocarcinoma cases, squamous cells carcinoma cases, and small cells carcinoma cases) and the covariance matrix of the coefficients.

#### B. Genotype-smoking interaction

We evaluated genotype-smoking interactions using a likelihood ratio test to compare the following two models:

- *Logit(LC) = α_0_+β×SNP+ζ×smoking status+γ×covariates* ,
- *Logit(LC) = α_0_+β×SNP+ζ×smoking status+θ×SNP×smoking status+γ×covariates*.

For polymorphisms showing the presence of a genotype-smoking interaction in the association with lung cancer, we fitted a model for the excess odds ratio of smoking (EOR) [54] in order to separate the contribution of total exposure and intensity in the interaction with the polymorphism. Specifically, we fitted the following 3-parameter linear-exponential model which described the OR in terms of continuous pack-years (*d*) and continuous cigarettes per day (*n*): *OR(d,n) = 1+β×d×g(n)*, where *β* is the EOR at *g(n)* = 1, i.e., the EOR/pack-year, and *g(.)* is a function that describes the influence of changing cigarettes per day on the strength of the lung cancer and pack-years association. Based on an empirical evaluation, we used a two parameter form for *g(.)*, where *g(n) = exp{ϕ_1_×ln(n)+ϕ_2_×ln(n)^2^}*. The component, *β×g(n)*, describes the EOR per pack-year and its variation with cigarettes per day and thus the influence of the delivery rate, i.e., increasing cigarettes per day and decreasing duration of exposure. We expanded this model to incorporate genotype (*s*, where *s* = 1 and 0 denote the variant and wild type forms, respectively), using: *OR(s,d,n) = exp(αs)×[1+β_s_×d×g_s_(n)]*, where the subscripts denote separate parameters for each genotype. We fitted the model to data on never and current smokers (including subjects who quit smoking less than two years before the study) and on never and former smokers (subjects who quit smoking more than two years before the study), and used likelihood ratio tests to compare homogeneity of the effects of pack-years, i.e., *β_1_ = β_0_*, and/or smoking intensity, i.e., *ϕ_s = 1,1_ = ϕ_s = 0,1_* and *ϕ_s = 1,2_ = ϕ_s = 0,2_*.

#### C. Joint SNPs

We analyzed multiple SNPs jointly to test whether the overall lung cancer risk was determined by the combined action of multiple SNPs within the same gene and/or of multiple genes within the same pathway, even if each SNP may have had only a modest effect size individually.

#### c1

Under the assumption that the effect on lung cancer of each SNP was *cumulative*, we implemented the following model:
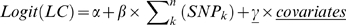
(1)where *k = 1*, *…*, *n* represents a collection of SNPs belonging to the same gene or a collection of SNPs belonging to genes in the same pathway (e.g. phase I, *n* = 25 i.e. all SNPs were grouped together). *SNP_k_* = 0 for the homozygote most common allele, *SNP_k_* = 1 for the heterozygote allele, and *SNP_k_* = 2 for the homozygote minor allele. *β* is the regression coefficient for the cumulative number of variants *Σ_k_^n^ (SNP_k_)*. We estimated the overall risk of lung cancer associated with each selected group of *n* SNPs by computing *OR = exp(β)* in the overall population, in never smokers, and in ever smokers separately. We estimated smoking-genotype interaction using the likelihood ratio test. Note that in this model we do not assume nor infer a risk direction for each minor allele. This approach will be powerful if minor alleles for all SNPs have effects in the same direction, but there may be loss of power if minor alleles for some SNPs affect lung cancer risk in opposite directions and their contribution to the overall risk cancels with each other.

#### c2

For all genes represented in our data by two or more SNPs, we computed paired linkage disequilibrium (LD) using the Haploview software and carried out haplotype analysis using the haplo.stats R-package.

#### D. Gene Expression

We evaluated to the extent possible, the effect of polymorphisms *SNP_k_^G^* from a given gene *G* on the gene expression of the same gene *G*, and specifically the effect related to lung cancer. We first estimated the overall effect of each group of SNPs (*SNP_k_^G^*) on lung cancer according to the additive model
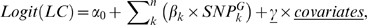
(2)where *β_k_* are the *n* regression coefficients for the *n* SNPs in *G*. Second, we used the *β_k_* estimated from equation (2) to compute the overall effect of each group of polymorphisms *SNP_k_^G^* on the change of gene expression of *G* (*Exp_G_*) by solving the following logistic regression:
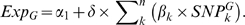
(3)According to equation (3), *δ>0* indicates an increase and *δ<0* a decrease in the gene expression of the gene *G*, due to the overall effect of the polymorphisms *SNP_k_^G^* on lung cancer. Basically, we used the SNPs regression score for lung cancer and verified whether it was positively or negatively associated with gene expression in non tumor tissue samples from a subgroup of cases. Note that since we lack gene expression data from healthy controls because no fresh frozen lung tissue samples can be collected from healthy people, we cannot measure directly the association between gene expression and lung cancer risk. Combining equation (2) and (3) instead, we are able to obtain such information. The described gene expression analysis was performed overall and, separately, among never smokers, former smokers, and current smokers.

#### E. Multiple testing and *a priori* knowledge considerations

We considered significant those results with a p-value less than (or equal to) 0.01. This choice was a compromise between a more stringent Bonferroni-corrected p-value and the loss in power from getting the threshold for significance too low. In addition, we referred to results with p-value between 0.01 and 0.05 as nominally significant, and considered them as notable when consistent across different analyses. Given the number of tested hypotheses in the single SNP analyses (25 tests corresponding to the 25 SNPs for the single SNP analysis and 5 tests when SNPs were grouped by genes) we took multiple testing into account. Our approach to multiple testing was informed by the selection strategy for the Phase I genes selected. Of note, each of the genes included has substantial mechanistic and at least some population data which support an association with lung cancer, as we have described in the introduction. We recognize that quantifying this *a priori* knowledge for each SNP is challenging, because of the heterogeneity of results in the literature and because most results actually refer to genes and not to our specific SNPs. In order to incorporate the effect of both multiple testing and *a priori* knowledge considerations, we computed the False Positive Report Probability (FPRP) [55] to characterize the noteworthiness for all the significant and nominally significant results from single SNP analyses for a range of prior probabilities. The statistical power to detect the measured OR given a type I error rate of 0.05 was computed by means of the QUANTO software (http://hydra.usc.edu/gxe).

## Supporting Information

Figure S1 Genes coverage for EPHX1, CYP1B1 and CYP1A1/A2
(0.68 MB DOC)

Figure S2 Results for linkage disequilibrium and haplotype analyses in CYP1A1 and CYP1A2.
(0.04 MB DOC)

Table S1 Associations between SNPs and lung cancer overall and major histology subtypes.
(0.79 MB DOC)

Table S2 Joint SNP analysis.
(0.05 MB DOC)

Table S3 Haplotype and three-marker moving window analyses in EPHX1.
(0.14 MB DOC)

Table S4 Gene expression and SNP correlation analysis.
(0.04 MB DOC)

Text S1 Results for linkage disequilibrium and haplotype analyses in EPHX1 and CYP1B1.
(0.03 MB DOC)
