# Supplementary material for: Phase I Metabolic Genes and Risk of Lung Cancer: Multiple Polymorphisms and mRNA Expression
Source: PLoS One. 2009 May 21;4(5):e5652. doi: 10.1371/journal.pone.0005652 (PMC2682568; doi:10.1371/journal.pone.0005652)
Supplement: Figure S1 — Genes coverage for EPHX1, CYP1B1 and CYP1A1/A2 (0.68 MB DOC) [file pone.0005652.s001.doc]

**Supplemental Figure 1. Genes coverage for EPHX1, CYP1B1 and CYP1A1/A2.**

Gene coverage from 19 of the selected SNPs in our study (see Table 1 from main text) in *EPHX1* (Figure S1A), *CYP1B1* (Figure S1B), and *CYP1A1/A2* (Figure S1C) genes. SNPs shown in Figures S1A, S1B, and S1C and their relative linkage disequilibrium estimates are obtained from the available data on the Caucasian population from the HapMap database. In addition to these 19 SNPs, our study included the following 4 SNPs which were not part of the HapMap database: *EPHX1* rs2854445, *EPHX1* rs34143170, *CYP1B1* rs163077, and *CYP1A1* rs2606345. Red circles indicate SNPs selected for this study (continuous circles) and SNPs in LD with them (dashed circles). Green circles indicate that the corresponding SNP has been also previously studied in association with lung cancer in other candidate genes studies. Blue circles indicate SNPs investigated in previous studies (continuous circles) or SNPs in LD with them (dashed circles), but not in the present study.

**Figure S1A.**

____________________________________EPHX1___________________________________

**Figure S1B.**

____________________________________CYP1B1____________________________________

**Figure S1C.**

____________CYP1A1_______________________________________CYP1A2_____________
