# Supplementary material for: Phase I Metabolic Genes and Risk of Lung Cancer: Multiple Polymorphisms and mRNA Expression
Source: PLoS One. 2009 May 21;4(5):e5652. doi: 10.1371/journal.pone.0005652 (PMC2682568; doi:10.1371/journal.pone.0005652)
Supplement: Figure S2 — Results for linkage disequilibrium and haplotype analyses in CYP1A1 and CYP1A2. (0.04 MB DOC) [file pone.0005652.s002.doc]

**Supplemental Figure S2.** **Results for linkage disequilibrium and haplotype analyses in CYP1A1 and CYP1A2.**

_____*CYP1A1*_________ *CYP1A2____*


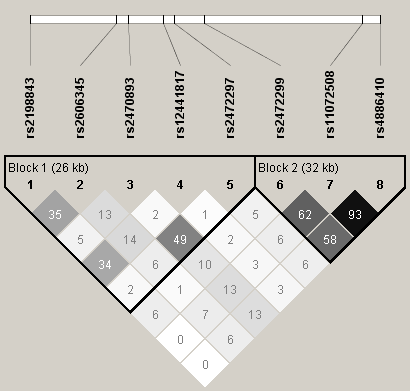


Figure S2 shows the LD analysis for the 8 SNPs in the15q24.1 region (*CYP1A1* and *CYP1A2* genes). Color scheme: r^2^ = 0, white; 0 < r^2^ < 1, shades of grey; r^2^ = 1, black. For each SNPs pair the r^2^ value is reported in the corresponding diamond. The presence of SNPs associated with lung cancer that are in low (r^2^ ≤ 0.1) and high (r^2^ > 0.6) pairwise LD suggests there may be independent gene effects on lung cancer risk at the chr15q24.1 CYP locus. For instance, *CYP1A2* rs11072508 and *CYP1A2* rs4886410 were in low LD (r^2^ = 0.07) with *CYP1A1* rs2606345 and all three SNPs were associated with lung cancer with a protecting trend in never smokers and a risk trend in ever smokers.

The haplotype analysis didn’t detect significant results, although the *CYP1A1* haplotype *GTAAA* among the never smokers (freq = 0.07) and the haplotype *CGGGG* among ever smokers (freq = 0.03) were nominally significantly associated with lung cancer (score = 2.30, p-value = 0.021; score = 2.05, p-value = 0.040; respectively).
