# Supplementary material for: Phase I Metabolic Genes and Risk of Lung Cancer: Multiple Polymorphisms and mRNA Expression
Source: PLoS One. 2009 May 21;4(5):e5652. doi: 10.1371/journal.pone.0005652 (PMC2682568; doi:10.1371/journal.pone.0005652)
Supplement: Table S1 — Associations between SNPs and lung cancer overall and major histology subtypes. (0.79 MB DOC) [file pone.0005652.s003.doc]

**Supplemental Table S1. Associations between SNPs and lung cancer overall and major histology subtypes.**

Results from logistic regression analysis of association between lung cancer and SNPs, in the overall population and by histology subtypes. ORs were adjusted for age, sex, area, cigarette per day, total pack-years, years since quit. Red color indicates p-value < 0.01, blue color p-value < 0.05, green color p-value < 0.1.

|  |  |  |  | **All controls and all cases** | |  |  |  |  |
| --- | --- | --- | --- | --- | --- | --- | --- | --- | --- |
| *Gene* | rs# | SNP Freq | Control | Case | Test | OR | CI1 | CI2 | P-value |
| *EPHX1* | rs2854455 | AA = | 1144 | 1095 | 1) Trend | 1 | 0.89 | 1.13 | 9.83E-01 |
|  |  | AB = | 736 | 657 | 2) 0=AA, 1=AB | 0.98 | 0.83 | 1.14 | 7.56E-01 |
|  |  | BB = | 139 | 113 | 3) 0=AA, 1=BB | 1.05 | 0.77 | 1.44 | 7.36E-01 |
|  |  | AB+BB = | 875 | 770 | 4) 0=AA, 1=AB+BB | 0.99 | 0.85 | 1.15 | 8.62E-01 |
| *G001S001* | ephx1_20 |  |  |  | 6) |  |  |  |  |
| *EPHX1* | rs3766934 | AA = | 1653 | 1514 | 1) Trend | 1.04 | 0.87 | 1.23 | 6.93E-01 |
|  |  | AB = | 354 | 321 | 2) 0=AA, 1=AB | 0.96 | 0.79 | 1.16 | 6.49E-01 |
|  |  | BB = | 21 | 29 | 3) 0=AA, 1=BB | 1.77 | 0.9 | 3.48 | 9.67E-02 |
|  |  | AB+BB = | 375 | 350 | 4) 0=AA, 1=AB+BB | 1 | 0.82 | 1.2 | 9.62E-01 |
| *G001S002* | ephx1_19 |  |  |  | 6) |  |  |  |  |
| *EPHX1* | rs2292566 | AA = | 1511 | 1370 | 1) Trend | 1.03 | 0.89 | 1.2 | 6.73E-01 |
|  |  | AB = | 485 | 464 | 2) 0=AA, 1=AB | 1.02 | 0.86 | 1.22 | 7.87E-01 |
|  |  | BB = | 38 | 34 | 3) 0=AA, 1=BB | 1.12 | 0.64 | 1.97 | 6.83E-01 |
|  |  | AB+BB = | 523 | 498 | 4) 0=AA, 1=AB+BB | 1.03 | 0.87 | 1.22 | 7.26E-01 |
| *G001S003* | ephx1_11 |  |  |  | 6) |  |  |  |  |
| *EPHX1* | rs2260863 | AA = | 912 | 884 | 1) Trend | 0.99 | 0.89 | 1.11 | 9.07E-01 |
|  |  | AB = | 911 | 778 | 2) 0=AA, 1=AB | 0.92 | 0.78 | 1.07 | 2.78E-01 |
|  |  | BB = | 206 | 202 | 3) 0=AA, 1=BB | 1.08 | 0.84 | 1.39 | 5.58E-01 |
|  |  | AB+BB = | 1117 | 980 | 4) 0=AA, 1=AB+BB | 0.95 | 0.82 | 1.1 | 4.63E-01 |
| *G001S004* | ephx1_10 |  |  |  | 6) |  |  |  |  |
| *EPHX1* | rs2234922 | AA = | 1316 | 1240 | 1) Trend | 0.95 | 0.83 | 1.09 | 4.48E-01 |
|  |  | AB = | 628 | 567 | 2) 0=AA, 1=AB | 1 | 0.85 | 1.17 | 9.96E-01 |
|  |  | BB = | 82 | 51 | 3) 0=AA, 1=BB | 0.75 | 0.49 | 1.14 | 1.75E-01 |
|  |  | AB+BB = | 710 | 618 | 4) 0=AA, 1=AB+BB | 0.97 | 0.83 | 1.14 | 7.20E-01 |
| *G001S005* | ephx1_01 |  |  |  | 6) |  |  |  |  |
| *EPHX1* | rs34143170 | AA = | 1791 | 1643 | 1) Trend | 0.99 | 0.79 | 1.23 | 8.99E-01 |
|  |  | AB = | 235 | 216 | 2) 0=AA, 1=AB | 1.02 | 0.8 | 1.28 | 8.96E-01 |
|  |  | BB = | 4 | 3 | 3) 0=AA, 1=BB | 0.45 | 0.09 | 2.29 | 3.34E-01 |
|  |  | AB+BB = | 239 | 219 | 4) 0=AA, 1=AB+BB | 1 | 0.79 | 1.26 | 9.94E-01 |
| *G001S006* | ephx1_24 |  |  |  | 6) |  |  |  |  |
| *EPHX1* | rs2292568 | AA = | 1852 | 1657 | 1) Trend | 1.22 | 0.95 | 1.56 | 1.20E-01 |
|  |  | AB = | 156 | 190 | 2) 0=AA, 1=AB | 1.28 | 0.99 | 1.66 | 6.46E-02 |
|  |  | BB = | 7 | 3 | 3) 0=AA, 1=BB | 0.59 | 0.12 | 2.92 | 5.16E-01 |
|  |  | AB+BB = | 163 | 193 | 4) 0=AA, 1=AB+BB | 1.25 | 0.97 | 1.62 | 8.42E-02 |
| *G001S007* | ephx1_13 |  |  |  | 6) |  |  |  |  |
| *EPHX1* | rs1051741 | AA = | 1631 | 1520 | 1) Trend | 1.01 | 0.85 | 1.21 | 8.73E-01 |
|  |  | AB = | 375 | 333 | 2) 0=AA, 1=AB | 1.04 | 0.86 | 1.26 | 6.82E-01 |
|  |  | BB = | 20 | 13 | 3) 0=AA, 1=BB | 0.81 | 0.37 | 1.79 | 6.07E-01 |
|  |  | AB+BB = | 395 | 346 | 4) 0=AA, 1=AB+BB | 1.03 | 0.85 | 1.24 | 7.65E-01 |
|  |  |  |  |  |  |  |  |  |  |
| *CYP1B1* | rs163077 | AA = | 1168 | 1087 | 1) Trend | 1.03 | 0.91 | 1.16 | 6.86E-01 |
|  |  | AB = | 728 | 668 | 2) 0=AA, 1=AB | 1.02 | 0.87 | 1.2 | 7.88E-01 |
|  |  | BB = | 129 | 107 | 3) 0=AA, 1=BB | 1.06 | 0.77 | 1.45 | 7.23E-01 |
|  |  | AB+BB = | 857 | 775 | 4) 0=AA, 1=AB+BB | 1.03 | 0.88 | 1.19 | 7.27E-01 |
| *G002S002* | fam82a_02 |  |  |  | 6) |  |  |  |  |
| *CYP1B1* | rs9341266 | AA = | 1798 | 1665 | 1) Trend | 0.86 | 0.69 | 1.08 | 2.08E-01 |
|  |  | AB = | 222 | 199 | 2) 0=AA, 1=AB | 0.93 | 0.73 | 1.18 | 5.47E-01 |
|  |  | BB = | 12 | 4 | 3) 0=AA, 1=BB | 0.23 | 0.06 | 0.92 | 3.82E-02 |
|  |  | AB+BB = | 234 | 203 | 4) 0=AA, 1=AB+BB | 0.89 | 0.71 | 1.13 | 3.50E-01 |
| *G002S003* | cyp1b1_59 |  |  |  | 6) |  |  |  |  |
| *CYP1B1* | rs162562 | AA = | 1446 | 1321 | 1) Trend | 1.07 | 0.93 | 1.23 | 3.73E-01 |
|  |  | AB = | 533 | 488 | 2) 0=AA, 1=AB | 1.03 | 0.87 | 1.22 | 7.15E-01 |
|  |  | BB = | 52 | 58 | 3) 0=AA, 1=BB | 1.28 | 0.82 | 1.99 | 2.77E-01 |
|  |  | AB+BB = | 585 | 546 | 4) 0=AA, 1=AB+BB | 1.05 | 0.9 | 1.24 | 5.23E-01 |
| *G002S004* | cyp1b1_31 |  |  |  | 6) |  |  |  |  |
| *CYP1B1* | rs1800440 | AA = | 1296 | 1188 | 1) Trend | 1.02 | 0.9 | 1.16 | 7.09E-01 |
|  |  | AB = | 654 | 580 | 2) 0=AA, 1=AB | 0.96 | 0.82 | 1.13 | 6.12E-01 |
|  |  | BB = | 82 | 97 | 3) 0=AA, 1=BB | 1.24 | 0.87 | 1.77 | 2.35E-01 |
|  |  | AB+BB = | 736 | 677 | 4) 0=AA, 1=AB+BB | 0.99 | 0.85 | 1.16 | 9.11E-01 |
| *G002S005* | cyp1b1_07 |  |  |  | 6) |  |  |  |  |
| *CYP1B1* | rs162557 | AA = | 1399 | 1272 | 1) Trend | 1.08 | 0.94 | 1.24 | 2.56E-01 |
|  |  | AB = | 575 | 529 | 2) 0=AA, 1=AB | 1.04 | 0.89 | 1.23 | 6.13E-01 |
|  |  | BB = | 59 | 67 | 3) 0=AA, 1=BB | 1.32 | 0.87 | 2 | 1.86E-01 |
|  |  | AB+BB = | 634 | 596 | 4) 0=AA, 1=AB+BB | 1.07 | 0.91 | 1.25 | 4.04E-01 |
| *G002S006* | cyp1b1_42 |  |  |  | 6) |  |  |  |  |
| *CYP1B1* | rs162556 | AA = | 621 | 517 | 1) Trend | 1.1 | 0.99 | 1.22 | 8.81E-02 |
|  |  | AB = | 1002 | 944 | 2) 0=AA, 1=AB | 1.1 | 0.92 | 1.3 | 2.92E-01 |
|  |  | BB = | 400 | 395 | 3) 0=AA, 1=BB | 1.2 | 0.97 | 1.49 | 9.08E-02 |
|  |  | AB+BB = | 1402 | 1339 | 4) 0=AA, 1=AB+BB | 1.13 | 0.96 | 1.33 | 1.51E-01 |
| *G002S007* | cyp1b1_27 |  |  |  | 6) |  |  |  |  |
| *CYP1B1* | rs10175368 | AA = | 1056 | 983 | 1) Trend | 0.89 | 0.79 | 1 | 5.51E-02 |
|  |  | AB = | 790 | 754 | 2) 0=AA, 1=AB | 0.97 | 0.83 | 1.13 | 6.84E-01 |
|  |  | BB = | 176 | 124 | 3) 0=AA, 1=BB | 0.7 | 0.52 | 0.93 | 1.39E-02 |
|  |  | AB+BB = | 966 | 878 | 4) 0=AA, 1=AB+BB | 0.92 | 0.79 | 1.06 | 2.55E-01 |
| *G002S008* | cyp1b1_18 |  |  |  | 6) |  |  |  |  |
| *CYP1A1* | rs2198843 | AA = | 1402 | 1267 | 1) Trend | 0.99 | 0.86 | 1.13 | 8.49E-01 |
|  |  | AB = | 562 | 522 | 2) 0=AA, 1=AB | 0.91 | 0.77 | 1.08 | 2.79E-01 |
|  |  | BB = | 64 | 78 | 3) 0=AA, 1=BB | 1.23 | 0.83 | 1.82 | 3.08E-01 |
|  |  | AB+BB = | 626 | 600 | 4) 0=AA, 1=AB+BB | 0.94 | 0.81 | 1.11 | 4.84E-01 |
| *G003S001* | cyp1a1_78 |  |  |  | 6) |  |  |  |  |
| *CYP1A1* | rs26066345 | AA = | 841 | 797 | 1) Trend | 0.99 | 0.89 | 1.1 | 8.74E-01 |
|  |  | AB = | 922 | 823 | 2) 0=AA, 1=AB | 0.93 | 0.8 | 1.09 | 3.94E-01 |
|  |  | BB = | 265 | 246 | 3) 0=AA, 1=BB | 1.03 | 0.81 | 1.3 | 8.09E-01 |
|  |  | AB+BB = | 1187 | 1069 | 4) 0=AA, 1=AB+BB | 0.95 | 0.82 | 1.11 | 5.40E-01 |
| *G003S002* | cyp1a1_14 |  |  |  | 6) |  |  |  |  |
| *CYP1A1* | rs2470893 | AA = | 1283 | 1170 | 1) Trend | 1.04 | 0.92 | 1.18 | 5.38E-01 |
|  |  | AB = | 664 | 603 | 2) 0=AA, 1=AB | 0.97 | 0.82 | 1.13 | 6.79E-01 |
|  |  | BB = | 80 | 91 | 3) 0=AA, 1=BB | 1.33 | 0.92 | 1.92 | 1.28E-01 |
|  |  | AB+BB = | 744 | 694 | 4) 0=AA, 1=AB+BB | 1 | 0.86 | 1.17 | 9.58E-01 |
| *G003S003* | cyp1a1_114 |  |  |  | 6) |  |  |  |  |
| *CYP1A1* | rs12441817 | AA = | 1723 | 1537 | 1) Trend | 1.12 | 0.93 | 1.34 | 2.29E-01 |
|  |  | AB = | 294 | 302 | 2) 0=AA, 1=AB | 1.06 | 0.87 | 1.3 | 5.67E-01 |
|  |  | BB = | 13 | 23 | 3) 0=AA, 1=BB | 1.87 | 0.86 | 4.1 | 1.16E-01 |
|  |  | AB+BB = | 307 | 325 | 4) 0=AA, 1=AB+BB | 1.1 | 0.9 | 1.34 | 3.67E-01 |
| *G003S004* | cyp1a1_113 |  |  |  | 6) |  |  |  |  |
| *CYP1A1* | rs2472297 | AA = | 1579 | 1456 | 1) Trend | 0.99 | 0.84 | 1.16 | 8.67E-01 |
|  |  | AB = | 426 | 383 | 2) 0=AA, 1=AB | 0.95 | 0.79 | 1.14 | 5.69E-01 |
|  |  | BB = | 21 | 24 | 3) 0=AA, 1=BB | 1.31 | 0.66 | 2.6 | 4.44E-01 |
|  |  | AB+BB = | 447 | 407 | 4) 0=AA, 1=AB+BB | 0.97 | 0.81 | 1.15 | 6.96E-01 |
| *G003S005* | cyp1a1_115 |  |  |  | 6) |  |  |  |  |
| *CYP1A1* | rs2472299 | AA = | 958 | 877 | 1) Trend | 1.02 | 0.91 | 1.14 | 7.63E-01 |
|  |  | AB = | 848 | 780 | 2) 0=AA, 1=AB | 0.98 | 0.84 | 1.15 | 8.23E-01 |
|  |  | BB = | 229 | 209 | 3) 0=AA, 1=BB | 1.07 | 0.84 | 1.37 | 5.84E-01 |
|  |  | AB+BB = | 1077 | 989 | 4) 0=AA, 1=AB+BB | 1 | 0.86 | 1.16 | 1.00E+00 |
| *G003S006* | cyp1a1_81 |  |  |  | 6) |  |  |  |  |
| *CYP1A2* | rs11072508 | AA = | 762 | 688 | 1) Trend | 1.07 | 0.96 | 1.19 | 2.34E-01 |
|  |  | AB = | 963 | 864 | 2) 0=AA, 1=AB | 1.01 | 0.86 | 1.19 | 9.13E-01 |
|  |  | BB = | 306 | 309 | 3) 0=AA, 1=BB | 1.17 | 0.94 | 1.47 | 1.62E-01 |
|  |  | AB+BB = | 1269 | 1173 | 4) 0=AA, 1=AB+BB | 1.05 | 0.9 | 1.22 | 5.54E-01 |
| *G003S007* | cyp1a2_79 |  |  |  | 6) |  |  |  |  |
| *CYP1A2* | rs4886410 | AA = | 770 | 701 | 1) Trend | 1.06 | 0.96 | 1.18 | 2.63E-01 |
|  |  | AB = | 965 | 867 | 2) 0=AA, 1=AB | 1 | 0.85 | 1.18 | 9.67E-01 |
|  |  | BB = | 295 | 298 | 3) 0=AA, 1=BB | 1.17 | 0.93 | 1.47 | 1.74E-01 |
|  |  | AB+BB = | 1260 | 1165 | 4) 0=AA, 1=AB+BB | 1.04 | 0.89 | 1.21 | 6.08E-01 |
| *G003S008* | csk_01 |  |  |  | 6) |  |  |  |  |
| *CYP2A6* | rs1801272 | AA = | 1855 | 1756 | 1) Trend | 0.72 | 0.54 | 0.96 | 2.58E-02 |
|  |  | AB = | 160 | 101 | 2) 0=AA, 1=AB | 0.74 | 0.55 | 1 | 5.24E-02 |
|  |  | BB = | 4 | 2 | 3) 0=AA, 1=BB | 0.26 | 0.04 | 1.94 | 1.90E-01 |
|  |  | AB+BB = | 164 | 103 | 4) 0=AA, 1=AB+BB | 0.73 | 0.54 | 0.98 | 3.54E-02 |
| *G004S001* | cyp2a6_01 |  |  |  | 6) |  |  |  |  |
| *MPO* | rs2333227 | AA = | 1123 | 1055 | 1) Trend | 0.94 | 0.84 | 1.06 | 3.48E-01 |
|  |  | AB = | 750 | 677 | 2) 0=AA, 1=AB | 0.88 | 0.76 | 1.03 | 1.23E-01 |
|  |  | BB = | 138 | 129 | 3) 0=AA, 1=BB | 1 | 0.74 | 1.35 | 9.80E-01 |
|  |  | AB+BB = | 888 | 806 | 4) 0=AA, 1=AB+BB | 0.9 | 0.78 | 1.05 | 1.74E-01 |

| **All controls and adenocarcinoma cases** | | | | | | | | | |
| --- | --- | --- | --- | --- | --- | --- | --- | --- | --- |
| Gene | rs# | SNP Freq | Control | Case | Comparison | OR | CI1 | CI2 | P-value |
| *EPHX1* | rs2854455 | AA = | 1144 | 450 | 1) Trend | 1.02 | 0.88 | 1.19 | 7.82E-01 |
|  |  | AB = | 736 | 276 | 2) 0=AA, 1=AB | 1.02 | 0.84 | 1.24 | 8.10E-01 |
|  |  | BB = | 139 | 47 | 3) 0=AA, 1=BB | 1.04 | 0.71 | 1.53 | 8.52E-01 |
|  |  | AB+BB = | 875 | 323 | 4) 0=AA, 1=AB+BB | 1.03 | 0.85 | 1.24 | 7.85E-01 |
| *G001S001* | ephx1_20 |  |  |  | 6) |  |  |  |  |
| *EPHX1* | rs3766934 | AA = | 1653 | 645 | 1) Trend | 0.97 | 0.78 | 1.2 | 7.46E-01 |
|  |  | AB = | 354 | 112 | 2) 0=AA, 1=AB | 0.82 | 0.64 | 1.06 | 1.34E-01 |
|  |  | BB = | 21 | 16 | 3) 0=AA, 1=BB | 2.05 | 0.97 | 4.33 | 5.94E-02 |
|  |  | AB+BB = | 375 | 128 | 4) 0=AA, 1=AB+BB | 0.89 | 0.7 | 1.13 | 3.39E-01 |
| *G001S002* | ephx1_19 |  |  |  | 6) |  |  |  |  |
| *EPHX1* | rs2292566 | AA = | 1511 | 556 | 1) Trend | 1.09 | 0.91 | 1.32 | 3.44E-01 |
|  |  | AB = | 485 | 202 | 2) 0=AA, 1=AB | 1.09 | 0.88 | 1.35 | 4.26E-01 |
|  |  | BB = | 38 | 16 | 3) 0=AA, 1=BB | 1.22 | 0.63 | 2.37 | 5.61E-01 |
|  |  | AB+BB = | 523 | 218 | 4) 0=AA, 1=AB+BB | 1.1 | 0.89 | 1.35 | 3.73E-01 |
| *G001S003* | ephx1_11 |  |  |  | 6) |  |  |  |  |
| *EPHX1* | rs2260863 | AA = | 912 | 360 | 1) Trend | 1 | 0.87 | 1.15 | 9.52E-01 |
|  |  | AB = | 911 | 327 | 2) 0=AA, 1=AB | 0.91 | 0.75 | 1.11 | 3.54E-01 |
|  |  | BB = | 206 | 88 | 3) 0=AA, 1=BB | 1.12 | 0.82 | 1.53 | 4.82E-01 |
|  |  | AB+BB = | 1117 | 415 | 4) 0=AA, 1=AB+BB | 0.95 | 0.79 | 1.14 | 5.77E-01 |
| *G001S004* | ephx1_10 |  |  |  | 6) |  |  |  |  |
| *EPHX1* | rs2234922 | AA = | 1316 | 520 | 1) Trend | 0.9 | 0.76 | 1.07 | 2.30E-01 |
|  |  | AB = | 628 | 228 | 2) 0=AA, 1=AB | 0.93 | 0.76 | 1.13 | 4.61E-01 |
|  |  | BB = | 82 | 22 | 3) 0=AA, 1=BB | 0.74 | 0.44 | 1.25 | 2.59E-01 |
|  |  | AB+BB = | 710 | 250 | 4) 0=AA, 1=AB+BB | 0.91 | 0.75 | 1.1 | 3.21E-01 |
| *G001S005* | ephx1_01 |  |  |  | 6) |  |  |  |  |
| *EPHX1* | rs34143170 | AA = | 1791 | 687 | 1) Trend | 0.92 | 0.69 | 1.23 | 5.89E-01 |
|  |  | AB = | 235 | 84 | 2) 0=AA, 1=AB | 0.98 | 0.73 | 1.31 | 8.71E-01 |
|  |  | BB = | 4 | 0 | 3) 0=AA, 1=BB | 0 | 0 | Inf | 9.59E-01 |
|  |  | AB+BB = | 239 | 84 | 4) 0=AA, 1=AB+BB | 0.95 | 0.71 | 1.27 | 7.31E-01 |
| *G001S006* | ephx1_24 |  |  |  | 6) |  |  |  |  |
| *EPHX1* | rs2292568 | AA = | 1852 | 680 | 1) Trend | 1.38 | 1.03 | 1.85 | 3.21E-02 |
|  |  | AB = | 156 | 86 | 2) 0=AA, 1=AB | 1.48 | 1.09 | 2.01 | 1.30E-02 |
|  |  | BB = | 7 | 1 | 3) 0=AA, 1=BB | 0.41 | 0.04 | 4.43 | 4.65E-01 |
|  |  | AB+BB = | 163 | 87 | 4) 0=AA, 1=AB+BB | 1.44 | 1.06 | 1.96 | 1.89E-02 |
| *G001S007* | ephx1_13 |  |  |  | 6) |  |  |  |  |
| *EPHX1* | rs1051741 | AA = | 1631 | 639 | 1) Trend | 0.94 | 0.75 | 1.17 | 5.75E-01 |
|  |  | AB = | 375 | 131 | 2) 0=AA, 1=AB | 0.95 | 0.75 | 1.21 | 7.02E-01 |
|  |  | BB = | 20 | 5 | 3) 0=AA, 1=BB | 0.75 | 0.27 | 2.13 | 5.91E-01 |
|  |  | AB+BB = | 395 | 136 | 4) 0=AA, 1=AB+BB | 0.94 | 0.75 | 1.2 | 6.34E-01 |
|  |  |  |  |  |  |  |  |  |  |
| *CYP1B1* | rs163077 | AA = | 1168 | 441 | 1) Trend | 1.09 | 0.94 | 1.26 | 2.64E-01 |
|  |  | AB = | 728 | 282 | 2) 0=AA, 1=AB | 1.09 | 0.9 | 1.32 | 4.01E-01 |
|  |  | BB = | 129 | 50 | 3) 0=AA, 1=BB | 1.19 | 0.81 | 1.75 | 3.74E-01 |
|  |  | AB+BB = | 857 | 332 | 4) 0=AA, 1=AB+BB | 1.1 | 0.92 | 1.32 | 3.07E-01 |
| *G002S002* | fam82a_02 |  |  |  | 6) |  |  |  |  |
| *CYP1B1* | rs9341266 | AA = | 1798 | 701 | 1) Trend | 0.74 | 0.55 | 0.99 | 4.58E-02 |
|  |  | AB = | 222 | 72 | 2) 0=AA, 1=AB | 0.8 | 0.59 | 1.09 | 1.57E-01 |
|  |  | BB = | 12 | 1 | 3) 0=AA, 1=BB | 0.14 | 0.01 | 1.24 | 7.73E-02 |
|  |  | AB+BB = | 234 | 73 | 4) 0=AA, 1=AB+BB | 0.76 | 0.56 | 1.04 | 8.37E-02 |
| *G002S003* | cyp1b1_59 |  |  |  | 6) |  |  |  |  |
| *CYP1B1* | rs162562 | AA = | 1446 | 543 | 1) Trend | 1.06 | 0.89 | 1.26 | 5.34E-01 |
|  |  | AB = | 533 | 209 | 2) 0=AA, 1=AB | 1.05 | 0.85 | 1.29 | 6.73E-01 |
|  |  | BB = | 52 | 21 | 3) 0=AA, 1=BB | 1.17 | 0.66 | 2.06 | 5.91E-01 |
|  |  | AB+BB = | 585 | 230 | 4) 0=AA, 1=AB+BB | 1.06 | 0.86 | 1.29 | 5.93E-01 |
| *G002S004* | cyp1b1_31 |  |  |  | 6) |  |  |  |  |
| *CYP1B1* | rs1800440 | AA = | 1296 | 485 | 1) Trend | 1.07 | 0.92 | 1.26 | 3.65E-01 |
|  |  | AB = | 654 | 240 | 2) 0=AA, 1=AB | 0.96 | 0.79 | 1.17 | 7.01E-01 |
|  |  | BB = | 82 | 47 | 3) 0=AA, 1=BB | 1.49 | 0.98 | 2.25 | 6.03E-02 |
|  |  | AB+BB = | 736 | 287 | 4) 0=AA, 1=AB+BB | 1.02 | 0.84 | 1.23 | 8.29E-01 |
| *G002S005* | cyp1b1_07 |  |  |  | 6) |  |  |  |  |
| *CYP1B1* | rs162557 | AA = | 1399 | 527 | 1) Trend | 1.05 | 0.89 | 1.25 | 5.57E-01 |
|  |  | AB = | 575 | 221 | 2) 0=AA, 1=AB | 1.02 | 0.83 | 1.25 | 8.61E-01 |
|  |  | BB = | 59 | 25 | 3) 0=AA, 1=BB | 1.25 | 0.74 | 2.11 | 4.08E-01 |
|  |  | AB+BB = | 634 | 246 | 4) 0=AA, 1=AB+BB | 1.04 | 0.85 | 1.26 | 7.06E-01 |
| *G002S006* | cyp1b1_42 |  |  |  | 6) |  |  |  |  |
| *CYP1B1* | rs162556 | AA = | 621 | 205 | 1) Trend | 1.16 | 1.01 | 1.32 | 3.12E-02 |
|  |  | AB = | 1002 | 391 | 2) 0=AA, 1=AB | 1.15 | 0.92 | 1.42 | 2.19E-01 |
|  |  | BB = | 400 | 172 | 3) 0=AA, 1=BB | 1.34 | 1.03 | 1.74 | 3.10E-02 |
|  |  | AB+BB = | 1402 | 563 | 4) 0=AA, 1=AB+BB | 1.2 | 0.98 | 1.47 | 8.36E-02 |
| *G002S007* | cyp1b1_27 |  |  |  | 6) |  |  |  |  |
| *CYP1B1* | rs10175368 | AA = | 1056 | 430 | 1) Trend | 0.8 | 0.69 | 0.93 | 2.90E-03 |
|  |  | AB = | 790 | 297 | 2) 0=AA, 1=AB | 0.87 | 0.71 | 1.05 | 1.42E-01 |
|  |  | BB = | 176 | 45 | 3) 0=AA, 1=BB | 0.55 | 0.38 | 0.81 | 2.26E-03 |
|  |  | AB+BB = | 966 | 342 | 4) 0=AA, 1=AB+BB | 0.81 | 0.67 | 0.97 | 2.25E-02 |
| *G002S008* | cyp1b1_18 |  |  |  | 6) |  |  |  |  |
| *CYP1A1* | rs2198843 | AA = | 1402 | 529 | 1) Trend | 0.97 | 0.82 | 1.15 | 7.05E-01 |
|  |  | AB = | 562 | 213 | 2) 0=AA, 1=AB | 0.89 | 0.73 | 1.1 | 2.78E-01 |
|  |  | BB = | 64 | 31 | 3) 0=AA, 1=BB | 1.2 | 0.74 | 1.95 | 4.62E-01 |
|  |  | AB+BB = | 626 | 244 | 4) 0=AA, 1=AB+BB | 0.92 | 0.76 | 1.12 | 4.27E-01 |
| *G003S001* | cyp1a1_78 |  |  |  | 6) |  |  |  |  |
| *CYP1A1* | rs26066345 | AA = | 841 | 332 | 1) Trend | 1.03 | 0.91 | 1.18 | 6.19E-01 |
|  |  | AB = | 922 | 324 | 2) 0=AA, 1=AB | 0.89 | 0.73 | 1.08 | 2.26E-01 |
|  |  | BB = | 265 | 118 | 3) 0=AA, 1=BB | 1.19 | 0.9 | 1.57 | 2.23E-01 |
|  |  | AB+BB = | 1187 | 442 | 4) 0=AA, 1=AB+BB | 0.95 | 0.79 | 1.14 | 5.91E-01 |
| *G003S002* | cyp1a1_14 |  |  |  | 6) |  |  |  |  |
| *CYP1A1* | rs2470893 | AA = | 1283 | 496 | 1) Trend | 1.01 | 0.86 | 1.19 | 8.88E-01 |
|  |  | AB = | 664 | 232 | 2) 0=AA, 1=AB | 0.9 | 0.74 | 1.11 | 3.30E-01 |
|  |  | BB = | 80 | 43 | 3) 0=AA, 1=BB | 1.34 | 0.87 | 2.06 | 1.79E-01 |
|  |  | AB+BB = | 744 | 275 | 4) 0=AA, 1=AB+BB | 0.95 | 0.79 | 1.15 | 6.23E-01 |
| *G003S003* | cyp1a1_114 |  |  |  | 6) |  |  |  |  |
| *CYP1A1* | rs12441817 | AA = | 1723 | 631 | 1) Trend | 1.17 | 0.93 | 1.47 | 1.74E-01 |
|  |  | AB = | 294 | 131 | 2) 0=AA, 1=AB | 1.12 | 0.88 | 1.44 | 3.57E-01 |
|  |  | BB = | 13 | 8 | 3) 0=AA, 1=BB | 1.9 | 0.72 | 5 | 1.92E-01 |
|  |  | AB+BB = | 307 | 139 | 4) 0=AA, 1=AB+BB | 1.16 | 0.9 | 1.48 | 2.48E-01 |
| *G003S004* | cyp1a1_113 |  |  |  | 6) |  |  |  |  |
| *CYP1A1* | rs2472297 | AA = | 1579 | 605 | 1) Trend | 0.95 | 0.78 | 1.17 | 6.56E-01 |
|  |  | AB = | 426 | 157 | 2) 0=AA, 1=AB | 0.92 | 0.73 | 1.16 | 4.83E-01 |
|  |  | BB = | 21 | 11 | 3) 0=AA, 1=BB | 1.17 | 0.52 | 2.65 | 7.02E-01 |
|  |  | AB+BB = | 447 | 168 | 4) 0=AA, 1=AB+BB | 0.93 | 0.75 | 1.17 | 5.51E-01 |
| *G003S005* | cyp1a1_115 |  |  |  | 6) |  |  |  |  |
| *CYP1A1* | rs2472299 | AA = | 958 | 364 | 1) Trend | 1.04 | 0.91 | 1.19 | 5.67E-01 |
|  |  | AB = | 848 | 321 | 2) 0=AA, 1=AB | 1.01 | 0.83 | 1.22 | 9.55E-01 |
|  |  | BB = | 229 | 89 | 3) 0=AA, 1=BB | 1.12 | 0.83 | 1.52 | 4.65E-01 |
|  |  | AB+BB = | 1077 | 410 | 4) 0=AA, 1=AB+BB | 1.03 | 0.86 | 1.24 | 7.64E-01 |
| *G003S006* | cyp1a1_81 |  |  |  | 6) |  |  |  |  |
| *CYP1A2* | rs11072508 | AA = | 762 | 278 | 1) Trend | 1.14 | 1 | 1.3 | 5.46E-02 |
|  |  | AB = | 963 | 360 | 2) 0=AA, 1=AB | 1.08 | 0.88 | 1.32 | 4.56E-01 |
|  |  | BB = | 306 | 133 | 3) 0=AA, 1=BB | 1.33 | 1.01 | 1.75 | 4.12E-02 |
|  |  | AB+BB = | 1269 | 493 | 4) 0=AA, 1=AB+BB | 1.14 | 0.94 | 1.38 | 1.85E-01 |
| *G003S007* | cyp1a2_79 |  |  |  | 6) |  |  |  |  |
| *CYP1A2* | rs4886410 | AA = | 770 | 287 | 1) Trend | 1.13 | 0.99 | 1.29 | 8.16E-02 |
|  |  | AB = | 965 | 360 | 2) 0=AA, 1=AB | 1.05 | 0.86 | 1.29 | 6.12E-01 |
|  |  | BB = | 295 | 127 | 3) 0=AA, 1=BB | 1.31 | 1 | 1.73 | 5.36E-02 |
|  |  | AB+BB = | 1260 | 487 | 4) 0=AA, 1=AB+BB | 1.11 | 0.92 | 1.34 | 2.78E-01 |
| *G003S008* | csk_01 |  |  |  | 6) |  |  |  |  |
| *CYP2A6* | rs1801272 | AA = | 1855 | 720 | 1) Trend | 0.83 | 0.59 | 1.18 | 3.01E-01 |
|  |  | AB = | 160 | 48 | 2) 0=AA, 1=AB | 0.84 | 0.58 | 1.21 | 3.36E-01 |
|  |  | BB = | 4 | 2 | 3) 0=AA, 1=BB | 0.68 | 0.1 | 4.73 | 6.93E-01 |
|  |  | AB+BB = | 164 | 50 | 4) 0=AA, 1=AB+BB | 0.83 | 0.58 | 1.19 | 3.11E-01 |
| *G004S001* | cyp2a6_01 |  |  |  | 6) |  |  |  |  |
| *MPO* | rs2333227 | AA = | 1123 | 423 | 1) Trend | 1.04 | 0.9 | 1.2 | 6.21E-01 |
|  |  | AB = | 750 | 274 | 2) 0=AA, 1=AB | 0.91 | 0.74 | 1.1 | 3.20E-01 |
|  |  | BB = | 138 | 72 | 3) 0=AA, 1=BB | 1.31 | 0.93 | 1.84 | 1.21E-01 |
|  |  | AB+BB = | 888 | 346 | 4) 0=AA, 1=AB+BB | 0.97 | 0.8 | 1.16 | 7.25E-01 |

|  | **All controls and squamous cell carcinoma cases** | | | | | | | |  |
| --- | --- | --- | --- | --- | --- | --- | --- | --- | --- |
| Gene | rs# | SNP Freq | Control | Case | Comparison | OR | CI1 | CI2 | P-value |
| *EPHX1* | rs2854455 | AA = | 1144 | 288 | 1) Trend | 1.08 | 0.89 | 1.31 | 4.32E-01 |
|  |  | AB = | 736 | 157 | 2) 0=AA, 1=AB | 0.93 | 0.72 | 1.21 | 6.02E-01 |
|  |  | BB = | 139 | 37 | 3) 0=AA, 1=BB | 1.48 | 0.93 | 2.37 | 9.71E-02 |
|  |  | AB+BB = | 875 | 194 | 4) 0=AA, 1=AB+BB | 1.01 | 0.79 | 1.28 | 9.40E-01 |
| *G001S001* | ephx1_20 |  |  |  | 6) |  |  |  |  |
| *EPHX1* | rs3766934 | AA = | 1653 | 385 | 1) Trend | 1.16 | 0.88 | 1.52 | 3.06E-01 |
|  |  | AB = | 354 | 92 | 2) 0=AA, 1=AB | 1.12 | 0.83 | 1.52 | 4.51E-01 |
|  |  | BB = | 21 | 5 | 3) 0=AA, 1=BB | 1.75 | 0.51 | 6.02 | 3.77E-01 |
|  |  | AB+BB = | 375 | 97 | 4) 0=AA, 1=AB+BB | 1.14 | 0.85 | 1.54 | 3.69E-01 |
| *G001S002* | ephx1_19 |  |  |  | 6) |  |  |  |  |
| *EPHX1* | rs2292566 | AA = | 1511 | 366 | 1) Trend | 0.88 | 0.68 | 1.13 | 3.05E-01 |
|  |  | AB = | 485 | 109 | 2) 0=AA, 1=AB | 0.85 | 0.64 | 1.13 | 2.61E-01 |
|  |  | BB = | 38 | 8 | 3) 0=AA, 1=BB | 0.94 | 0.36 | 2.41 | 8.90E-01 |
|  |  | AB+BB = | 523 | 117 | 4) 0=AA, 1=AB+BB | 0.86 | 0.65 | 1.13 | 2.67E-01 |
| *G001S003* | ephx1_11 |  |  |  | 6) |  |  |  |  |
| *EPHX1* | rs2260863 | AA = | 912 | 241 | 1) Trend | 0.95 | 0.79 | 1.14 | 5.70E-01 |
|  |  | AB = | 911 | 192 | 2) 0=AA, 1=AB | 0.85 | 0.66 | 1.09 | 2.03E-01 |
|  |  | BB = | 206 | 50 | 3) 0=AA, 1=BB | 1.03 | 0.68 | 1.56 | 8.90E-01 |
|  |  | AB+BB = | 1117 | 242 | 4) 0=AA, 1=AB+BB | 0.88 | 0.7 | 1.12 | 2.95E-01 |
| *G001S004* | ephx1_10 |  |  |  | 6) |  |  |  |  |
| *EPHX1* | rs2234922 | AA = | 1316 | 309 | 1) Trend | 1.05 | 0.85 | 1.31 | 6.37E-01 |
|  |  | AB = | 628 | 159 | 2) 0=AA, 1=AB | 1.14 | 0.89 | 1.48 | 2.98E-01 |
|  |  | BB = | 82 | 12 | 3) 0=AA, 1=BB | 0.79 | 0.39 | 1.63 | 5.29E-01 |
|  |  | AB+BB = | 710 | 171 | 4) 0=AA, 1=AB+BB | 1.11 | 0.87 | 1.42 | 4.11E-01 |
| *G001S005* | ephx1_01 |  |  |  | 6) |  |  |  |  |
| *EPHX1* | rs34143170 | AA = | 1791 | 418 | 1) Trend | 1.25 | 0.89 | 1.76 | 1.98E-01 |
|  |  | AB = | 235 | 64 | 2) 0=AA, 1=AB | 1.34 | 0.94 | 1.91 | 1.10E-01 |
|  |  | BB = | 4 | 1 | 3) 0=AA, 1=BB | 0.43 | 0.04 | 4.58 | 4.85E-01 |
|  |  | AB+BB = | 239 | 65 | 4) 0=AA, 1=AB+BB | 1.3 | 0.92 | 1.86 | 1.42E-01 |
| *G001S006* | ephx1_24 |  |  |  | 6) |  |  |  |  |
| *EPHX1* | rs2292568 | AA = | 1852 | 432 | 1) Trend | 1.14 | 0.76 | 1.71 | 5.30E-01 |
|  |  | AB = | 156 | 45 | 2) 0=AA, 1=AB | 1.2 | 0.79 | 1.83 | 3.84E-01 |
|  |  | BB = | 7 | 0 | 3) 0=AA, 1=BB | 0 | 0 | Inf | 9.78E-01 |
|  |  | AB+BB = | 163 | 45 | 4) 0=AA, 1=AB+BB | 1.17 | 0.78 | 1.78 | 4.48E-01 |
| *G001S007* | ephx1_13 |  |  |  | 6) |  |  |  |  |
| *EPHX1* | rs1051741 | AA = | 1631 | 383 | 1) Trend | 1.06 | 0.8 | 1.4 | 6.85E-01 |
|  |  | AB = | 375 | 99 | 2) 0=AA, 1=AB | 1.16 | 0.87 | 1.57 | 3.11E-01 |
|  |  | BB = | 20 | 0 | 3) 0=AA, 1=BB | 0 | 0 | Inf | 9.76E-01 |
|  |  | AB+BB = | 395 | 99 | 4) 0=AA, 1=AB+BB | 1.12 | 0.83 | 1.5 | 4.58E-01 |
|  |  |  |  |  |  |  |  |  |  |
| *CYP1B1* | rs163077 | AA = | 1168 | 285 | 1) Trend | 1.01 | 0.83 | 1.23 | 8.97E-01 |
|  |  | AB = | 728 | 168 | 2) 0=AA, 1=AB | 0.93 | 0.72 | 1.2 | 5.72E-01 |
|  |  | BB = | 129 | 28 | 3) 0=AA, 1=BB | 1.24 | 0.74 | 2.07 | 4.16E-01 |
|  |  | AB+BB = | 857 | 196 | 4) 0=AA, 1=AB+BB | 0.97 | 0.76 | 1.23 | 7.88E-01 |
| *G002S002* | fam82a_02 |  |  |  | 6) |  |  |  |  |
| *CYP1B1* | rs9341266 | AA = | 1798 | 429 | 1) Trend | 0.91 | 0.64 | 1.3 | 6.13E-01 |
|  |  | AB = | 222 | 52 | 2) 0=AA, 1=AB | 1.01 | 0.69 | 1.48 | 9.58E-01 |
|  |  | BB = | 12 | 2 | 3) 0=AA, 1=BB | 0.31 | 0.05 | 1.79 | 1.88E-01 |
|  |  | AB+BB = | 234 | 54 | 4) 0=AA, 1=AB+BB | 0.96 | 0.66 | 1.4 | 8.18E-01 |
| *G002S003* | cyp1b1_59 |  |  |  | 6) |  |  |  |  |
| *CYP1B1* | rs162562 | AA = | 1446 | 353 | 1) Trend | 0.95 | 0.76 | 1.19 | 6.78E-01 |
|  |  | AB = | 533 | 112 | 2) 0=AA, 1=AB | 0.84 | 0.63 | 1.11 | 2.10E-01 |
|  |  | BB = | 52 | 18 | 3) 0=AA, 1=BB | 1.37 | 0.7 | 2.65 | 3.55E-01 |
|  |  | AB+BB = | 585 | 130 | 4) 0=AA, 1=AB+BB | 0.89 | 0.68 | 1.15 | 3.71E-01 |
| *G002S004* | cyp1b1_31 |  |  |  | 6) |  |  |  |  |
| *CYP1B1* | rs1800440 | AA = | 1296 | 313 | 1) Trend | 0.97 | 0.79 | 1.2 | 8.04E-01 |
|  |  | AB = | 654 | 141 | 2) 0=AA, 1=AB | 0.85 | 0.65 | 1.1 | 2.06E-01 |
|  |  | BB = | 82 | 27 | 3) 0=AA, 1=BB | 1.33 | 0.77 | 2.28 | 3.09E-01 |
|  |  | AB+BB = | 736 | 168 | 4) 0=AA, 1=AB+BB | 0.9 | 0.7 | 1.15 | 3.98E-01 |
| *G002S005* | cyp1b1_07 |  |  |  | 6) |  |  |  |  |
| *CYP1B1* | rs162557 | AA = | 1399 | 340 | 1) Trend | 0.97 | 0.78 | 1.21 | 8.11E-01 |
|  |  | AB = | 575 | 122 | 2) 0=AA, 1=AB | 0.86 | 0.66 | 1.13 | 2.77E-01 |
|  |  | BB = | 59 | 21 | 3) 0=AA, 1=BB | 1.37 | 0.73 | 2.56 | 3.29E-01 |
|  |  | AB+BB = | 634 | 143 | 4) 0=AA, 1=AB+BB | 0.91 | 0.7 | 1.18 | 4.68E-01 |
| *G002S006* | cyp1b1_42 |  |  |  | 6) |  |  |  |  |
| *CYP1B1* | rs162556 | AA = | 621 | 144 | 1) Trend | 1.01 | 0.85 | 1.2 | 8.81E-01 |
|  |  | AB = | 1002 | 237 | 2) 0=AA, 1=AB | 0.93 | 0.7 | 1.22 | 5.90E-01 |
|  |  | BB = | 400 | 99 | 3) 0=AA, 1=BB | 1.05 | 0.74 | 1.47 | 7.94E-01 |
|  |  | AB+BB = | 1402 | 336 | 4) 0=AA, 1=AB+BB | 0.96 | 0.74 | 1.24 | 7.57E-01 |
| *G002S007* | cyp1b1_27 |  |  |  | 6) |  |  |  |  |
| *CYP1B1* | rs10175368 | AA = | 1056 | 243 | 1) Trend | 0.99 | 0.83 | 1.19 | 9.38E-01 |
|  |  | AB = | 790 | 200 | 2) 0=AA, 1=AB | 1.09 | 0.85 | 1.4 | 4.82E-01 |
|  |  | BB = | 176 | 38 | 3) 0=AA, 1=BB | 0.86 | 0.55 | 1.34 | 4.94E-01 |
|  |  | AB+BB = | 966 | 238 | 4) 0=AA, 1=AB+BB | 1.05 | 0.83 | 1.33 | 6.98E-01 |
| *G002S008* | cyp1b1_18 |  |  |  | 6) |  |  |  |  |
| *CYP1A1* | rs2198843 | AA = | 1402 | 329 | 1) Trend | 1 | 0.81 | 1.24 | 9.94E-01 |
|  |  | AB = | 562 | 133 | 2) 0=AA, 1=AB | 0.96 | 0.73 | 1.25 | 7.40E-01 |
|  |  | BB = | 64 | 21 | 3) 0=AA, 1=BB | 1.13 | 0.62 | 2.06 | 6.99E-01 |
|  |  | AB+BB = | 626 | 154 | 4) 0=AA, 1=AB+BB | 0.98 | 0.76 | 1.26 | 8.49E-01 |
| *G003S001* | cyp1a1_78 |  |  |  | 6) |  |  |  |  |
| *CYP1A1* | rs26066345 | AA = | 841 | 219 | 1) Trend | 0.95 | 0.8 | 1.13 | 5.56E-01 |
|  |  | AB = | 922 | 201 | 2) 0=AA, 1=AB | 0.87 | 0.68 | 1.13 | 2.96E-01 |
|  |  | BB = | 265 | 61 | 3) 0=AA, 1=BB | 0.96 | 0.66 | 1.4 | 8.52E-01 |
|  |  | AB+BB = | 1187 | 262 | 4) 0=AA, 1=AB+BB | 0.89 | 0.7 | 1.13 | 3.54E-01 |
| *G003S002* | cyp1a1_14 |  |  |  | 6) |  |  |  |  |
| *CYP1A1* | rs2470893 | AA = | 1283 | 300 | 1) Trend | 1.09 | 0.89 | 1.34 | 3.93E-01 |
|  |  | AB = | 664 | 159 | 2) 0=AA, 1=AB | 1.1 | 0.85 | 1.42 | 4.60E-01 |
|  |  | BB = | 80 | 23 | 3) 0=AA, 1=BB | 1.17 | 0.66 | 2.11 | 5.88E-01 |
|  |  | AB+BB = | 744 | 182 | 4) 0=AA, 1=AB+BB | 1.11 | 0.87 | 1.42 | 4.06E-01 |
| *G003S003* | cyp1a1_114 |  |  |  | 6) |  |  |  |  |
| *CYP1A1* | rs12441817 | AA = | 1723 | 413 | 1) Trend | 1.02 | 0.74 | 1.39 | 9.20E-01 |
|  |  | AB = | 294 | 65 | 2) 0=AA, 1=AB | 0.94 | 0.67 | 1.31 | 7.02E-01 |
|  |  | BB = | 13 | 4 | 3) 0=AA, 1=BB | 2.58 | 0.62 | 10.76 | 1.95E-01 |
|  |  | AB+BB = | 307 | 69 | 4) 0=AA, 1=AB+BB | 0.97 | 0.7 | 1.36 | 8.76E-01 |
| *G003S004* | cyp1a1_113 |  |  |  | 6) |  |  |  |  |
| *CYP1A1* | rs2472297 | AA = | 1579 | 379 | 1) Trend | 0.96 | 0.74 | 1.25 | 7.61E-01 |
|  |  | AB = | 426 | 96 | 2) 0=AA, 1=AB | 0.92 | 0.68 | 1.23 | 5.63E-01 |
|  |  | BB = | 21 | 6 | 3) 0=AA, 1=BB | 1.33 | 0.44 | 3.99 | 6.12E-01 |
|  |  | AB+BB = | 447 | 102 | 4) 0=AA, 1=AB+BB | 0.93 | 0.7 | 1.24 | 6.45E-01 |
| *G003S005* | cyp1a1_115 |  |  |  | 6) |  |  |  |  |
| *CYP1A1* | rs2472299 | AA = | 958 | 217 | 1) Trend | 1.06 | 0.89 | 1.27 | 5.23E-01 |
|  |  | AB = | 848 | 207 | 2) 0=AA, 1=AB | 0.96 | 0.75 | 1.24 | 7.59E-01 |
|  |  | BB = | 229 | 58 | 3) 0=AA, 1=BB | 1.24 | 0.84 | 1.85 | 2.85E-01 |
|  |  | AB+BB = | 1077 | 265 | 4) 0=AA, 1=AB+BB | 1.01 | 0.8 | 1.28 | 9.28E-01 |
| *G003S006* | cyp1a1_81 |  |  |  | 6) |  |  |  |  |
| *CYP1A2* | rs11072508 | AA = | 762 | 170 | 1) Trend | 1.15 | 0.97 | 1.37 | 1.11E-01 |
|  |  | AB = | 963 | 225 | 2) 0=AA, 1=AB | 1.02 | 0.79 | 1.33 | 8.54E-01 |
|  |  | BB = | 306 | 85 | 3) 0=AA, 1=BB | 1.4 | 0.98 | 2 | 6.13E-02 |
|  |  | AB+BB = | 1269 | 310 | 4) 0=AA, 1=AB+BB | 1.11 | 0.86 | 1.41 | 4.24E-01 |
| *G003S007* | cyp1a2_79 |  |  |  | 6) |  |  |  |  |
| *CYP1A2* | rs4886410 | AA = | 770 | 172 | 1) Trend | 1.15 | 0.96 | 1.36 | 1.22E-01 |
|  |  | AB = | 965 | 226 | 2) 0=AA, 1=AB | 1.03 | 0.8 | 1.34 | 8.11E-01 |
|  |  | BB = | 295 | 83 | 3) 0=AA, 1=BB | 1.39 | 0.97 | 2 | 7.08E-02 |
|  |  | AB+BB = | 1260 | 309 | 4) 0=AA, 1=AB+BB | 1.11 | 0.87 | 1.42 | 4.17E-01 |
| *G003S008* | csk_01 |  |  |  | 6) |  |  |  |  |
| *CYP2A6* | rs1801272 | AA = | 1855 | 463 | 1) Trend | 0.47 | 0.27 | 0.81 | 7.37E-03 |
|  |  | AB = | 160 | 18 | 2) 0=AA, 1=AB | 0.48 | 0.27 | 0.86 | 1.28E-02 |
|  |  | BB = | 4 | 0 | 3) 0=AA, 1=BB | 0 | 0 | Inf | 9.82E-01 |
|  |  | AB+BB = | 164 | 18 | 4) 0=AA, 1=AB+BB | 0.47 | 0.27 | 0.83 | 8.85E-03 |
| *G004S001* | cyp2a6_01 |  |  |  | 6) |  |  |  |  |
| *MPO* | rs2333227 | AA = | 1123 | 273 | 1) Trend | 0.91 | 0.75 | 1.11 | 3.46E-01 |
|  |  | AB = | 750 | 181 | 2) 0=AA, 1=AB | 0.89 | 0.69 | 1.14 | 3.41E-01 |
|  |  | BB = | 138 | 27 | 3) 0=AA, 1=BB | 0.88 | 0.53 | 1.46 | 6.18E-01 |
|  |  | AB+BB = | 888 | 208 | 4) 0=AA, 1=AB+BB | 0.89 | 0.7 | 1.12 | 3.16E-01 |

| **All controls and small cell carcinoma cases** | | | | | | | | | |
| --- | --- | --- | --- | --- | --- | --- | --- | --- | --- |
| Gene | rs# | SNP Freq | Control | Case | Comparison | OR | CI1 | CI2 | P-value |
| *EPHX1* | rs2854455 | AA = | 1144 | 103 | 1) Trend | 1.12 | 0.85 | 1.47 | 4.38E-01 |
|  |  | AB = | 736 | 72 | 2) 0=AA, 1=AB | 1.18 | 0.83 | 1.67 | 3.66E-01 |
|  |  | BB = | 139 | 10 | 3) 0=AA, 1=BB | 1.11 | 0.53 | 2.33 | 7.89E-01 |
|  |  | AB+BB = | 875 | 82 | 4) 0=AA, 1=AB+BB | 1.17 | 0.83 | 1.64 | 3.70E-01 |
| *G001S001* | ephx1_20 |  |  |  | 6) |  |  |  |  |
| *EPHX1* | rs3766934 | AA = | 1653 | 146 | 1) Trend | 1.11 | 0.75 | 1.63 | 6.16E-01 |
|  |  | AB = | 354 | 36 | 2) 0=AA, 1=AB | 1.07 | 0.7 | 1.64 | 7.56E-01 |
|  |  | BB = | 21 | 2 | 3) 0=AA, 1=BB | 1.61 | 0.31 | 8.44 | 5.73E-01 |
|  |  | AB+BB = | 375 | 38 | 4) 0=AA, 1=AB+BB | 1.09 | 0.72 | 1.66 | 6.82E-01 |
| *G001S002* | ephx1_19 |  |  |  | 6) |  |  |  |  |
| *EPHX1* | rs2292566 | AA = | 1511 | 130 | 1) Trend | 1.18 | 0.84 | 1.66 | 3.39E-01 |
|  |  | AB = | 485 | 53 | 2) 0=AA, 1=AB | 1.24 | 0.85 | 1.8 | 2.63E-01 |
|  |  | BB = | 38 | 2 | 3) 0=AA, 1=BB | 0.94 | 0.2 | 4.36 | 9.37E-01 |
|  |  | AB+BB = | 523 | 55 | 4) 0=AA, 1=AB+BB | 1.22 | 0.85 | 1.77 | 2.84E-01 |
| *G001S003* | ephx1_11 |  |  |  | 6) |  |  |  |  |
| *EPHX1* | rs2260863 | AA = | 912 | 90 | 1) Trend | 0.94 | 0.72 | 1.22 | 6.21E-01 |
|  |  | AB = | 911 | 72 | 2) 0=AA, 1=AB | 0.8 | 0.56 | 1.14 | 2.15E-01 |
|  |  | BB = | 206 | 20 | 3) 0=AA, 1=BB | 1.06 | 0.6 | 1.88 | 8.44E-01 |
|  |  | AB+BB = | 1117 | 92 | 4) 0=AA, 1=AB+BB | 0.84 | 0.6 | 1.18 | 3.17E-01 |
| *G001S004* | ephx1_10 |  |  |  | 6) |  |  |  |  |
| *EPHX1* | rs2234922 | AA = | 1316 | 127 | 1) Trend | 0.89 | 0.65 | 1.21 | 4.44E-01 |
|  |  | AB = | 628 | 52 | 2) 0=AA, 1=AB | 0.91 | 0.63 | 1.32 | 6.14E-01 |
|  |  | BB = | 82 | 5 | 3) 0=AA, 1=BB | 0.71 | 0.26 | 1.95 | 5.06E-01 |
|  |  | AB+BB = | 710 | 57 | 4) 0=AA, 1=AB+BB | 0.89 | 0.62 | 1.27 | 5.13E-01 |
| *G001S005* | ephx1_01 |  |  |  | 6) |  |  |  |  |
| *EPHX1* | rs34143170 | AA = | 1791 | 164 | 1) Trend | 0.89 | 0.53 | 1.48 | 6.45E-01 |
|  |  | AB = | 235 | 19 | 2) 0=AA, 1=AB | 0.83 | 0.48 | 1.43 | 5.07E-01 |
|  |  | BB = | 4 | 1 | 3) 0=AA, 1=BB | 1.76 | 0.17 | 18.54 | 6.40E-01 |
|  |  | AB+BB = | 239 | 20 | 4) 0=AA, 1=AB+BB | 0.85 | 0.5 | 1.46 | 5.65E-01 |
| *G001S006* | ephx1_24 |  |  |  | 6) |  |  |  |  |
| *EPHX1* | rs2292568 | AA = | 1852 | 164 | 1) Trend | 1.23 | 0.71 | 2.13 | 4.69E-01 |
|  |  | AB = | 156 | 19 | 2) 0=AA, 1=AB | 1.28 | 0.73 | 2.26 | 3.89E-01 |
|  |  | BB = | 7 | 0 | 3) 0=AA, 1=BB | 0 | 0 | Inf | 9.81E-01 |
|  |  | AB+BB = | 163 | 19 | 4) 0=AA, 1=AB+BB | 1.26 | 0.72 | 2.22 | 4.22E-01 |
| *G001S007* | ephx1_13 |  |  |  | 6) |  |  |  |  |
| *EPHX1* | rs1051741 | AA = | 1631 | 153 | 1) Trend | 1.08 | 0.73 | 1.6 | 6.97E-01 |
|  |  | AB = | 375 | 28 | 2) 0=AA, 1=AB | 0.88 | 0.56 | 1.4 | 5.99E-01 |
|  |  | BB = | 20 | 4 | 3) 0=AA, 1=BB | 3.09 | 0.92 | 10.4 | 6.87E-02 |
|  |  | AB+BB = | 395 | 32 | 4) 0=AA, 1=AB+BB | 0.98 | 0.63 | 1.52 | 9.32E-01 |
|  |  |  |  |  |  |  |  |  |  |
| *CYP1B1* | rs163077 | AA = | 1168 | 113 | 1) Trend | 0.95 | 0.72 | 1.26 | 7.41E-01 |
|  |  | AB = | 728 | 62 | 2) 0=AA, 1=AB | 0.92 | 0.65 | 1.32 | 6.70E-01 |
|  |  | BB = | 129 | 10 | 3) 0=AA, 1=BB | 0.97 | 0.46 | 2.04 | 9.43E-01 |
|  |  | AB+BB = | 857 | 72 | 4) 0=AA, 1=AB+BB | 0.93 | 0.66 | 1.31 | 6.85E-01 |
| *G002S002* | fam82a_02 |  |  |  | 6) |  |  |  |  |
| *CYP1B1* | rs9341266 | AA = | 1798 | 164 | 1) Trend | 0.83 | 0.5 | 1.38 | 4.74E-01 |
|  |  | AB = | 222 | 20 | 2) 0=AA, 1=AB | 0.98 | 0.57 | 1.7 | 9.55E-01 |
|  |  | BB = | 12 | 0 | 3) 0=AA, 1=BB | 0 | 0 | Inf | 9.79E-01 |
|  |  | AB+BB = | 234 | 20 | 4) 0=AA, 1=AB+BB | 0.9 | 0.52 | 1.54 | 6.93E-01 |
| *G002S003* | cyp1b1_59 |  |  |  | 6) |  |  |  |  |
| *CYP1B1* | rs162562 | AA = | 1446 | 129 | 1) Trend | 1.08 | 0.78 | 1.5 | 6.32E-01 |
|  |  | AB = | 533 | 51 | 2) 0=AA, 1=AB | 1.09 | 0.75 | 1.59 | 6.57E-01 |
|  |  | BB = | 52 | 4 | 3) 0=AA, 1=BB | 1.14 | 0.37 | 3.54 | 8.20E-01 |
|  |  | AB+BB = | 585 | 55 | 4) 0=AA, 1=AB+BB | 1.09 | 0.76 | 1.58 | 6.35E-01 |
| *G002S004* | cyp1b1_31 |  |  |  | 6) |  |  |  |  |
| *CYP1B1* | rs1800440 | AA = | 1296 | 121 | 1) Trend | 0.92 | 0.68 | 1.24 | 5.93E-01 |
|  |  | AB = | 654 | 57 | 2) 0=AA, 1=AB | 0.88 | 0.61 | 1.27 | 4.97E-01 |
|  |  | BB = | 82 | 7 | 3) 0=AA, 1=BB | 0.98 | 0.41 | 2.34 | 9.60E-01 |
|  |  | AB+BB = | 736 | 64 | 4) 0=AA, 1=AB+BB | 0.89 | 0.63 | 1.26 | 5.20E-01 |
| *G002S005* | cyp1b1_07 |  |  |  | 6) |  |  |  |  |
| *CYP1B1* | rs162557 | AA = | 1399 | 127 | 1) Trend | 1.05 | 0.76 | 1.44 | 7.79E-01 |
|  |  | AB = | 575 | 54 | 2) 0=AA, 1=AB | 1.05 | 0.73 | 1.52 | 7.81E-01 |
|  |  | BB = | 59 | 4 | 3) 0=AA, 1=BB | 1.06 | 0.35 | 3.23 | 9.17E-01 |
|  |  | AB+BB = | 634 | 58 | 4) 0=AA, 1=AB+BB | 1.05 | 0.74 | 1.51 | 7.73E-01 |
| *G002S006* | cyp1b1_42 |  |  |  | 6) |  |  |  |  |
| *CYP1B1* | rs162556 | AA = | 621 | 48 | 1) Trend | 1.15 | 0.9 | 1.47 | 2.59E-01 |
|  |  | AB = | 1002 | 95 | 2) 0=AA, 1=AB | 1.16 | 0.78 | 1.73 | 4.67E-01 |
|  |  | BB = | 400 | 39 | 3) 0=AA, 1=BB | 1.32 | 0.81 | 2.16 | 2.64E-01 |
|  |  | AB+BB = | 1402 | 134 | 4) 0=AA, 1=AB+BB | 1.2 | 0.82 | 1.76 | 3.38E-01 |
| *G002S007* | cyp1b1_27 |  |  |  | 6) |  |  |  |  |
| *CYP1B1* | rs10175368 | AA = | 1056 | 96 | 1) Trend | 0.86 | 0.66 | 1.12 | 2.59E-01 |
|  |  | AB = | 790 | 74 | 2) 0=AA, 1=AB | 1 | 0.7 | 1.42 | 9.91E-01 |
|  |  | BB = | 176 | 12 | 3) 0=AA, 1=BB | 0.58 | 0.3 | 1.15 | 1.19E-01 |
|  |  | AB+BB = | 966 | 86 | 4) 0=AA, 1=AB+BB | 0.91 | 0.65 | 1.28 | 5.84E-01 |
| *G002S008* | cyp1b1_18 |  |  |  | 6) |  |  |  |  |
| *CYP1A1* | rs2198843 | AA = | 1402 | 133 | 1) Trend | 0.82 | 0.6 | 1.12 | 2.13E-01 |
|  |  | AB = | 562 | 44 | 2) 0=AA, 1=AB | 0.7 | 0.47 | 1.03 | 7.18E-02 |
|  |  | BB = | 64 | 8 | 3) 0=AA, 1=BB | 1.06 | 0.45 | 2.46 | 8.99E-01 |
|  |  | AB+BB = | 626 | 52 | 4) 0=AA, 1=AB+BB | 0.74 | 0.51 | 1.07 | 1.05E-01 |
| *G003S001* | cyp1a1_78 |  |  |  | 6) |  |  |  |  |
| *CYP1A1* | rs26066345 | AA = | 841 | 80 | 1) Trend | 0.93 | 0.73 | 1.2 | 5.87E-01 |
|  |  | AB = | 922 | 87 | 2) 0=AA, 1=AB | 1.04 | 0.73 | 1.48 | 8.44E-01 |
|  |  | BB = | 265 | 18 | 3) 0=AA, 1=BB | 0.78 | 0.44 | 1.39 | 3.99E-01 |
|  |  | AB+BB = | 1187 | 105 | 4) 0=AA, 1=AB+BB | 0.98 | 0.7 | 1.38 | 9.12E-01 |
| *G003S002* | cyp1a1_14 |  |  |  | 6) |  |  |  |  |
| *CYP1A1* | rs2470893 | AA = | 1283 | 113 | 1) Trend | 1.06 | 0.8 | 1.42 | 6.82E-01 |
|  |  | AB = | 664 | 64 | 2) 0=AA, 1=AB | 1.12 | 0.78 | 1.59 | 5.40E-01 |
|  |  | BB = | 80 | 8 | 3) 0=AA, 1=BB | 0.98 | 0.42 | 2.27 | 9.56E-01 |
|  |  | AB+BB = | 744 | 72 | 4) 0=AA, 1=AB+BB | 1.1 | 0.78 | 1.55 | 5.83E-01 |
| *G003S003* | cyp1a1_114 |  |  |  | 6) |  |  |  |  |
| *CYP1A1* | rs12441817 | AA = | 1723 | 152 | 1) Trend | 1.29 | 0.86 | 1.94 | 2.21E-01 |
|  |  | AB = | 294 | 28 | 2) 0=AA, 1=AB | 1.01 | 0.63 | 1.61 | 9.61E-01 |
|  |  | BB = | 13 | 4 | 3) 0=AA, 1=BB | 8.12 | 1.97 | 33.38 | 3.70E-03 |
|  |  | AB+BB = | 307 | 32 | 4) 0=AA, 1=AB+BB | 1.15 | 0.74 | 1.8 | 5.37E-01 |
| *G003S004* | cyp1a1_113 |  |  |  | 6) |  |  |  |  |
| *CYP1A1* | rs2472297 | AA = | 1579 | 141 | 1) Trend | 0.99 | 0.68 | 1.43 | 9.41E-01 |
|  |  | AB = | 426 | 42 | 2) 0=AA, 1=AB | 1.06 | 0.71 | 1.58 | 7.84E-01 |
|  |  | BB = | 21 | 1 | 3) 0=AA, 1=BB | 0.44 | 0.05 | 3.63 | 4.45E-01 |
|  |  | AB+BB = | 447 | 43 | 4) 0=AA, 1=AB+BB | 1.02 | 0.69 | 1.52 | 9.06E-01 |
| *G003S005* | cyp1a1_115 |  |  |  | 6) |  |  |  |  |
| *CYP1A1* | rs2472299 | AA = | 958 | 88 | 1) Trend | 1.02 | 0.79 | 1.32 | 8.64E-01 |
|  |  | AB = | 848 | 73 | 2) 0=AA, 1=AB | 0.87 | 0.61 | 1.25 | 4.57E-01 |
|  |  | BB = | 229 | 22 | 3) 0=AA, 1=BB | 1.23 | 0.71 | 2.15 | 4.60E-01 |
|  |  | AB+BB = | 1077 | 95 | 4) 0=AA, 1=AB+BB | 0.94 | 0.67 | 1.31 | 7.02E-01 |
| *G003S006* | cyp1a1_81 |  |  |  | 6) |  |  |  |  |
| *CYP1A2* | rs11072508 | AA = | 762 | 71 | 1) Trend | 1.09 | 0.85 | 1.39 | 4.82E-01 |
|  |  | AB = | 963 | 83 | 2) 0=AA, 1=AB | 0.99 | 0.69 | 1.43 | 9.58E-01 |
|  |  | BB = | 306 | 30 | 3) 0=AA, 1=BB | 1.26 | 0.76 | 2.09 | 3.69E-01 |
|  |  | AB+BB = | 1269 | 113 | 4) 0=AA, 1=AB+BB | 1.05 | 0.74 | 1.48 | 7.82E-01 |
| *G003S007* | cyp1a2_79 |  |  |  | 6) |  |  |  |  |
| *CYP1A2* | rs4886410 | AA = | 770 | 71 | 1) Trend | 1.1 | 0.86 | 1.4 | 4.62E-01 |
|  |  | AB = | 965 | 85 | 2) 0=AA, 1=AB | 1.03 | 0.71 | 1.49 | 8.73E-01 |
|  |  | BB = | 295 | 29 | 3) 0=AA, 1=BB | 1.25 | 0.75 | 2.09 | 3.96E-01 |
|  |  | AB+BB = | 1260 | 114 | 4) 0=AA, 1=AB+BB | 1.08 | 0.76 | 1.52 | 6.69E-01 |
| *G003S008* | csk_01 |  |  |  | 6) |  |  |  |  |
| *CYP2A6* | rs1801272 | AA = | 1855 | 170 | 1) Trend | 1.05 | 0.57 | 1.94 | 8.71E-01 |
|  |  | AB = | 160 | 14 | 2) 0=AA, 1=AB | 1.11 | 0.59 | 2.09 | 7.45E-01 |
|  |  | BB = | 4 | 0 | 3) 0=AA, 1=BB | 0 | 0 | Inf | 9.77E-01 |
|  |  | AB+BB = | 164 | 14 | 4) 0=AA, 1=AB+BB | 1.08 | 0.58 | 2.03 | 8.04E-01 |
| *G004S001* | cyp2a6_01 |  |  |  | 6) |  |  |  |  |
| *MPO* | rs2333227 | AA = | 1123 | 103 | 1) Trend | 0.86 | 0.66 | 1.13 | 2.89E-01 |
|  |  | AB = | 750 | 73 | 2) 0=AA, 1=AB | 0.95 | 0.67 | 1.35 | 7.92E-01 |
|  |  | BB = | 138 | 9 | 3) 0=AA, 1=BB | 0.59 | 0.27 | 1.27 | 1.76E-01 |
|  |  | AB+BB = | 888 | 82 | 4) 0=AA, 1=AB+BB | 0.9 | 0.64 | 1.25 | 5.20E-01 |
