# Supplementary material for: Phase I Metabolic Genes and Risk of Lung Cancer: Multiple Polymorphisms and mRNA Expression
Source: PLoS One. 2009 May 21;4(5):e5652. doi: 10.1371/journal.pone.0005652 (PMC2682568; doi:10.1371/journal.pone.0005652)
Supplement: Table S2 — Joint SNP analysis. (0.05 MB DOC) [file pone.0005652.s004.doc]

**Supplemental Table S2. Joint SNP analysis.**

Results from the multiple SNP analysis based on the cumulative number of variants, in the overall population and stratified by never/ever smoking status and by histology type. P-values (Wald Test) are reported in bold if less than 0.01 and in *italics* if between 0.01 and 0.5.

|  |  |  |  |  |  | **Never** |  |  |  |  | **Ever** |  |  |  |  |  |
| --- | --- | --- | --- | --- | --- | --- | --- | --- | --- | --- | --- | --- | --- | --- | --- | --- |
| SNPs Group  (N SNPs) | Contr | Case | OR (b) | 95% CI | P-val  Trend | Contr | Case | OR (a) | 95% CI | P-val  Trend | Contr | Case | OR (b) | 95% CI |  | LH  Ratio |
| **All subjects** |  |  |  |  |  |  |  |  |  |  |  |  |  |  |  |  |
| EPHX1 (8) | 1966 | 1810 | 1.02 | 0.97-1.07 | 0.526 | 636 | 136 | 1.02 | 0.90-1.16 | 0.696 | 1330 | 1674 | 1.02 | 0.96-1.08 | 0.625 | 0.924 |
| CYP1B1 (7) | 1989 | 1830 | 1.03 | 0.96-1.12 | 0.421 | 642 | 134 | 1.16 | 0.95-1.41 | 0.175 | 1347 | 1696 | 1.01 | 0.93-1.10 | 0.782 | 0.23 |
| CYP1A1/A2 (8) | 1991 | 1826 | 1.01 | 0.98-1.05 | 0.379 | 647 | 136 | 0.91 | 0.84-0.99 | *0.040* | 1344 | 1690 | 1.03 | 1.00-1.07 | *0.055* | **0.006** |
| **Adenocarcinomas** |  |  |  |  |  |  |  |  |  |  |  |  |  |  |  |  |
| EPHX1 (8) | 1966 | 753 | 1.00 | 0.92-1.09 | 0.948 | 636 | 96 | 1.00 | 0.82-1.22 | 0.890 | 1330 | 657 | 1.02 | 0.91-1.10 | 0.985 | 0.959 |
| CYP1B1 (7) | 1989 | 757 | 1.02 | 0.93-1.12 | 0.695 | 642 | 95 | 1.05 | 0.83-1.32 | 0.677 | 1347 | 662 | 1.01 | 0.91-1.13 | 0.820 | 0.809 |
| CYP1A1/A2 (8) | 1991 | 755 | 1.03 | 0.98-1.08 | 0.286 | 647 | 96 | 0.95 | 0.85-1.06 | 0.423 | 1344 | 659 | 1.04 | 0.99-1.10 | 0.090 | 0.107 |
| CYP1B1 (2)(*) | 2021 | 801 | 0.83 | 0.74-0.94 | **0.002** | 651 | 98 | 0.85 | 0.65-1.11 | 0.226 | 1370 | 703 | 0.83 | 0.73-0.95 | **0.008** | 0.912 |

(a) ORs were adjusted for age, sex, area.

(b) ORs were adjusted for age, sex, area, cigarette per day, total pack-years, years since quit.

(*) *CYP1B1/rs9341266* and *CYP1B1/rs10175368*
