# Supplementary material for: Phase I Metabolic Genes and Risk of Lung Cancer: Multiple Polymorphisms and mRNA Expression
Source: PLoS One. 2009 May 21;4(5):e5652. doi: 10.1371/journal.pone.0005652 (PMC2682568; doi:10.1371/journal.pone.0005652)
Supplement: Table S3 — Haplotype and three-marker moving window analyses in EPHX1. (0.14 MB DOC) [file pone.0005652.s005.doc]

**Supplemental Table S3. Haplotype and three-marker moving window analyses in EPHX1.**

Results for the haplotype analysis in EPHX1, based on all 8 SNPs together and on three SNPs at a time (three-marker moving window analysis).

| loc-1 | loc-2 | loc-3 | loc-4 | loc-5 | loc-6 | loc-7 | loc-8 | Freq | Score | P-value |
| --- | --- | --- | --- | --- | --- | --- | --- | --- | --- | --- |
| T | G | G | C | A | C | T | C | 0.01 | 2.590 | **0.010** |
| C | G | G | C | G | C | C | T | 0.01 | -2.441 | **0.015** |
| C | G | G | C | G | C | C | C | 0.02 | 1.589 | 0.112 |
| T | G | G | C | G | T | C | C | 0.01 | -1.351 | 0.177 |
| T | G | G | C | A | C | C | C | 0.24 | -0.945 | 0.345 |
| T | G | A | C | A | C | T | C | 0.02 | 0.869 | 0.385 |
| T | G | G | C | A | C | C | T | 0.02 | 0.823 | 0.410 |
| T | T | G | C | A | C | C | C | 0.03 | 0.715 | 0.475 |
| T | G | G | C | G | C | C | T | 0.02 | 0.671 | 0.502 |
| C | G | G | C | A | C | C | C | 0.05 | 0.645 | 0.519 |
| T | T | G | C | A | T | C | C | 0.02 | 0.570 | 0.569 |
| C | G | G | G | G | C | C | T | 0.01 | -0.408 | 0.683 |
| C | G | G | G | A | C | C | C | 0.10 | 0.337 | 0.736 |
| T | G | A | C | A | C | C | C | 0.09 | -0.297 | 0.766 |
| T | G | G | C | G | C | C | C | 0.05 | -0.268 | 0.789 |
| T | G | G | G | A | C | C | C | 0.14 | -0.265 | 0.791 |
| T | G | G | C | A | T | C | C | 0.01 | -0.242 | 0.809 |
| T | T | A | C | A | C | C | C | 0.01 | -0.231 | 0.818 |
| T | T | G | G | A | C | C | C | 0.02 | -0.222 | 0.824 |
| T | G | A | C | G | C | C | T | 0.01 | -0.214 | 0.831 |
| T | G | G | G | G | C | C | C | 0.01 | -0.118 | 0.906 |
| C | G | G | G | G | C | C | C | 0.02 | 0.060 | 0.952 |
|  |  |  |  |  |  |  |  |  |  |  |
| loc-1 | loc-2 | loc-3 |  |  |  |  |  | Freq | Score | P-value |
| C | G | A |  |  |  |  |  | 0.01 | -1.257 | 0.209 |
| T | T | G |  |  |  |  |  | 0.09 | 0.675 | 0.500 |
| T | G | G |  |  |  |  |  | 0.54 | -0.606 | 0.544 |
| T | G | A |  |  |  |  |  | 0.12 | 0.345 | 0.730 |
| C | G | G |  |  |  |  |  | 0.23 | 0.287 | 0.774 |
| T | T | A |  |  |  |  |  | 0.01 | 0.254 | 0.800 |
|  |  |  |  |  |  |  |  |  |  |  |
|  |  |  | loc-4 | loc-5 | loc-6 |  |  | Freq | Score | P-value |
|  |  |  | C | G | T |  |  | 0.02 | -1.747 | 0.081 |
|  |  |  | C | A | T |  |  | 0.04 | 0.750 | 0.453 |
|  |  |  | C | G | C |  |  | 0.13 | -0.348 | 0.728 |
|  |  |  | C | A | C |  |  | 0.49 | 0.341 | 0.733 |
|  |  |  | G | G | C |  |  | 0.04 | -0.180 | 0.857 |
|  |  |  | G | A | C |  |  | 0.28 | 0.025 | 0.980 |
|  |  |  |  |  |  |  |  |  |  |  |
| loc-1 |  |  |  |  |  | loc-7 | loc-8 | Freq | Score | P-value |
| C |  |  |  |  |  | T | C | 0.04 | 1.152 | 0.249 |
| T |  |  |  |  |  | C | C | 0.06 | -0.708 | 0.479 |
| C |  |  |  |  |  | C | T | 0.09 | -0.437 | 0.662 |
| C |  |  |  |  |  | C | C | 0.80 | -0.121 | 0.903 |
|  |  |  |  |  |  |  |  |  |  |  |
|  | loc-2 | loc-3 | loc-4 |  |  |  |  | Freq | Score | P-value |
|  | T | G | C |  |  |  |  | 0.07 | 0.521 | 0.602 |
|  | T | A | C |  |  |  |  | 0.01 | 0.423 | 0.672 |
|  | G | G | C |  |  |  |  | 0.47 | -0.288 | 0.774 |
|  | T | G | G |  |  |  |  | 0.02 | -0.130 | 0.897 |
|  | G | G | G |  |  |  |  | 0.30 | -0.061 | 0.951 |
|  | G | A | C |  |  |  |  | 0.13 | 0.018 | 0.985 |
|  |  |  |  |  |  |  |  |  |  |  |
|  |  |  |  | loc-5 | loc-6 | loc-7 |  | Freq | Score | P-value |
|  |  |  |  | G | T | C |  | 0.02 | -1.715 | 0.086 |
|  |  |  |  | A | C | T |  | 0.04 | 1.251 | 0.211 |
|  |  |  |  | A | T | C |  | 0.04 | 0.572 | 0.567 |
|  |  |  |  | A | C | C |  | 0.73 | -0.340 | 0.734 |
|  |  |  |  | G | C | C |  | 0.17 | -0.234 | 0.815 |
|  |  |  |  |  |  |  |  |  |  |  |
| loc-1 | loc-2 |  |  |  |  |  | loc-8 | Freq | Score | P-value |
| C | G |  |  |  |  |  | T | 0.03 | -1.885 | 0.059 |
| T | T |  |  |  |  |  | C | 0.08 | 1.058 | 0.290 |
| T | G |  |  |  |  |  | T | 0.06 | 1.047 | 0.295 |
| T | G |  |  |  |  |  | C | 0.60 | -0.957 | 0.339 |
| C | G |  |  |  |  |  | C | 0.22 | 0.921 | 0.357 |
| T | T |  |  |  |  |  | T | 0.01 | -0.685 | 0.493 |
|  |  |  |  |  |  |  |  |  |  |  |
|  |  | loc-3 | loc-4 | loc-5 |  |  |  | Freq | Score | P-value |
|  |  | G | C | G |  |  |  | 0.13 | -0.791 | 0.429 |
|  |  | G | C | A |  |  |  | 0.41 | 0.475 | 0.635 |
|  |  | G | G | G |  |  |  | 0.04 | -0.283 | 0.777 |
|  |  | A | C | A |  |  |  | 0.12 | 0.157 | 0.875 |
|  |  | A | C | G |  |  |  | 0.02 | -0.074 | 0.941 |
|  |  | G | G | A |  |  |  | 0.28 | 0.033 | 0.974 |
|  |  |  |  |  |  |  |  |  |  |  |
|  |  |  |  |  | loc-6 | loc-7 | loc-8 | Freq | Score | P-value |
|  |  |  |  |  | C | T | C | 0.04 | 1.152 | 0.249 |
|  |  |  |  |  | T | C | C | 0.06 | -0.708 | 0.479 |
|  |  |  |  |  | C | C | T | 0.09 | -0.437 | 0.662 |
|  |  |  |  |  | C | C | C | 0.80 | -0.121 | 0.903 |
