# Supplementary material for: Phase I Metabolic Genes and Risk of Lung Cancer: Multiple Polymorphisms and mRNA Expression
Source: PLoS One. 2009 May 21;4(5):e5652. doi: 10.1371/journal.pone.0005652 (PMC2682568; doi:10.1371/journal.pone.0005652)
Supplement: Table S4 — Gene expression and SNP correlation analysis. (0.04 MB DOC) [file pone.0005652.s006.doc]

**Supplemental Table S4. Gene expression and SNP correlation analysis.**

Results from the gene expression and SNP correlation analysis in 44 adenocarcinoma cases overall and stratified by never/former/current smoking status. P-values (Wald Test) are reported in bold if less than 0.01 and in *italics* if between 0.01 and 0.5.

|  | **All** |  |  | **Never** |  |  | **Former** |  |  | **Current** |  |  |
| --- | --- | --- | --- | --- | --- | --- | --- | --- | --- | --- | --- | --- |
| Gene | Cases | Coef δ | P-value | Cases | Coef δ | P-value | Cases | Coef | P-value | Cases | Coef δ | P-value |
| EPHX1 | 42* | -1.20 | 0.096 | 14 | -1.27 | 0.334 | 13* | -2.56 | *0.049* | 15 | -0.21 | 0.874 |
| CYP1B1 | 41* | 0.90 | 0.674 | 13* | -0.94 | 0.507 | 14* | 1.04 | 0.704 | 14* | 8.99 | **0.004** |
| CYP1A1/A2 | 44 | 0.57 | 0.556 | 14 | -1.51 | **0.007** | 15 | -0.28 | 0.450 | 15 | 4.95 | 0.078 |
| CYP2A6 | 44 | 0.18 | 0.589 | 14 | -0.50 | 0.226 | 15 | Inf | 1 | 15 | 1.05 | 0.095 |
| MPO1 | 43* | 1.30 | 0.570 | 14 | -0.59 | 0.921 | 14* | 2.29 | 0.256 | 15 | 0.81 | 0.896 |

***Number of samples may vary across groups due to missing genotype data.
