# Supplementary material for: Phase I Metabolic Genes and Risk of Lung Cancer: Multiple Polymorphisms and mRNA Expression
Source: PLoS One. 2009 May 21;4(5):e5652. doi: 10.1371/journal.pone.0005652 (PMC2682568; doi:10.1371/journal.pone.0005652)
Supplement: Text S1 — Results for linkage disequilibrium and haplotype analyses in EPHX1 and CYP1B1. (0.03 MB DOC) [file pone.0005652.s007.doc]

**Supplemental Text S1.** **Results for linkage disequilibrium and haplotype analyses in EPHX1 and CYP1B1.**

*EPHX1*

The 8 SNPs in *EPHX1* were in low LD (r^2^ ≤ 0.1 for most SNPs pairs, r^2^ = 0.43 for *EPHX1* rs2234922 and *EPHX1* rs1051741). As reported in the main text, two haplotypes were significantly associated with lung cancer in the overall population: *TGGCACTC* (freq = 0.009, score = 2.59, p-value = 0.010) and *CGGCGCCT* (freq = 0.012, score = -2.44, p-value = 0.015). Similarly among adenocarcinoma cases only: *TGGCACTC* (freq = 0.01, score = 2.64, p-value = 0.008) and *CGGCGCCT* (freq = 0.01, score = -2.27, p-value = 0.023).

*CYP1B1*

Most pairs from the 6 SNPs in *CYP1B1* were in low LD (r^2^ ≤ 0.1), two pairs (*CYP1B1* rs1800440 - *CYP1B1* rs162556 and *CYP1B1* rs162556 - *CYP1B1* rs10175368) were in medium-low LD (r^2^ =0.30) and *CYP1B1* rs162562 - *CYP1B1* rs162557 were in high LD (r^2^ = 0.84). The association between lung cancer and the haplotype *CAACTA* in adenocarcinoma cases only (freq = 0.03) was nominally significant (score = 2.02, p-value = 0.043).
